# Phylogenomic analysis provides insights into *MADS-box* and *TCP* gene diversification and floral development of the Asteraceae, supported by *de novo* genome and transcriptome sequences from dandelion (*Taraxacum officinale*)

**DOI:** 10.3389/fpls.2023.1198909

**Published:** 2023-06-21

**Authors:** Wei Xiong, Judith Risse, Lidija Berke, Tao Zhao, Henri van de Geest, Carla Oplaat, Marco Busscher, Julie Ferreira de Carvalho, Ingrid M. van der Meer, Koen J. F. Verhoeven, M. Eric Schranz, Kitty Vijverberg

**Affiliations:** ^1^ Biosystematics Group, Wageningen University and Research, Wageningen, Netherlands; ^2^ Bioinformatics Group, Wageningen University and Research, Wageningen, Netherlands; ^3^ Department of Terrestrial Ecology, Netherlands Institute of Ecology (NIOO-KNAW), Wageningen, Netherlands; ^4^ Bioscience, Wageningen University and Research, Wageningen, Netherlands

**Keywords:** Asteraceae, dandelion, *de novo* sequencing, floral development, *MADS-box* gene, phylogenomics, *TCP* gene

## Abstract

The Asteraceae is the largest angiosperm family with more than 25,000 species. Individual studies have shown that *MADS-box* and *TCP* transcription factors are regulators of the development and symmetry of flowers, contributing to their iconic flower-head (capitulum) and floret. However, a systematic study of *MADS-box* and *TCP* genes across the Asteraceae is lacking. We performed a comparative analysis of genome sequences of 33 angiosperm species including our *de novo* assembly of diploid sexual dandelion (*Taraxacum officinale*) and 11 other Asteraceae to investigate the lineage-specific evolution of *MADS-box* and *TCP* genes in the Asteraceae. We compared the phylogenomic results of *MADS-box* and *TCP* genes with their expression in *T. officinale* floral tissues at different developmental stages to demonstrate the regulation of genes with Asteraceae-specific attributes. Here, we show that *MADS-box MIKC* ^c^ and *TCP-CYCLOIDEA* (*CYC*) genes have expanded in the Asteraceae. The phylogenomic analysis identified *AGAMOUS*-like (*AG*-like: *SEEDSTICK* [*STK*]-like), *SEPALATA*-like (*SEP*3-like), and *TCP-PROLIFERATING CELL FACTOR* (*PCF*)-like copies with lineage-specific genomic contexts in the Asteraceae, Cichorioideae, or dandelion. Different expression patterns of some of these gene copies suggest functional divergence. We also confirm the presence and revisit the evolutionary history of previously named “*Asteraceae-Specific MADS-box* genes (*AS-MADS*).” Specifically, we identify non-Asteraceae homologs, indicating a more ancient origin of this gene clade. Syntenic relationships support that *AS-MADS* is paralogous to *FLOWERING LOCUS C* (*FLC*) as demonstrated by the shared ancient duplication of *FLC* and *SEP*3.

## 1 Introduction

The Asteraceae (Compositae) is the largest and most diverse families of angiosperms, with great ecological and economic importance. It contains ~25,000 species, which represents 10% of extant flowering plants (Mandel et al., 2019). The Asteraceae is subdivided into 16 subfamilies including two large crown-groups, the Asteroideae (e.g., sunflower and daisy) and Cichorioideae (e.g., lettuce and dandelion) (Chase et al., 2016; Stevens, 2017; Susanna et al., 2020). Members of the Asteraceae inhabit an incredible range of ecosystems varying in climates and landscapes on every continent (Smith and Richardson, 2010; Folk et al., 2020). Their global distribution makes them interesting targets to study various questions in ecology and evolution (Shen et al., 2021; Palazzesi et al., 2022). For humans, the Asteraceae is of considerable societal and economic value as ornamentals (e.g., *Gerbera* and *Chrysanthemum*), medicines (e.g., sweet wormwood and chamomile), and crops (e.g., sunflower and lettuce), including many well-known weedy species (e.g., groundsel and dandelion). Genome assemblies can facilitate the study of the molecular and evolutionary bases of ecological and economic traits. To date, most sequenced Asteraceae species are ornamentals and crops.

The unique floral and fruit traits of the Asteraceae, including the representative flower heads (capitula) and one-seeded dry fruits (cypsela) often with a hairy or scaly pappus, underlie much of the diversity and evolutionary and ecological success of the group (Panero and Funk, 2008; Mandel et al., 2019). The capitulum is one of the most iconic floral features of the Asteraceae, a highly compressed inflorescence with many closely packed flowers, named “florets”, that together resemble a flower (Elomaa et al., 2018). There are three major floret types in Asteraceae—disc (tube), ray (two- three-lobed), and ligulate (five-lobed) (Anderberg et al., 2007)—which are discriminatory to the subfamilies, particularly the Asteroideae, characterized by disc florets ± one or more rows of ray florets, and the Cichorioideae, characterized by ligulate florets (Carlquist, 1976). In addition, the pappus, a highly modified calyx (Vijverberg et al., 2021), is another striking characteristic of the Asteraceae. It assists in seed dispersal and can protect against herbivores and aid in water uptake to facilitate germination (Carlquist, 1976; Stuessy and Garver, 1996; Jana and Mukherjee, 2012). Understanding the genetic basis of capitulum formation and floral and fruit characteristics is, therefore, of large interest to understanding the evolutionary success of the Asteraceae.

Whole-genome duplications (WGDs) have likely played a critical role in boosting the diversity of the Asteraceae (Barker et al., 2008), similar to other angiosperm lineages (Ohno, 1970; De Bodt et al., 2005; Magadum et al., 2013). In the Asteraceae, two paleopolyploid events occurred preceding their major radiation (Barker et al., 2016), and more recent WGDs occurred in major tribes and subfamilies (Huang et al., 2016; Shen et al., 2021). After WGDs, the additional gene copies may retain their original function (redundant copies) or undergo sub- and/or neo-functionalization (Panchy et al., 2016). Moreover, genes in a new genomic context (i.e., gene transposition) may result in a novel (*cis*) gene regulation (Ilic et al., 2003; Langham et al., 2004; Lockton and Gaut, 2005). Among the most important regulators of floral organ determination and development are the *MADS-box* and *TCP* transcription factors. Polyploidization has resulted in expanded *MADS-box* and *TCP* gene families. These expansions have been shown to contribute to the evolution of the capitulum, floral and fruit characteristics in the Asteraceae in different studies (see below).

In this study, we further examine *MADS-box* and *TCP* gene families to study their evolution, genomic context, and expression. The *MADS-box* gene family consists of two major clades: Type I and Type II. Type I genes have a conserved N-terminal MADS DNA binding domain (M). Type II genes contain an M-domain, a less conserved Intervening domain (I), a conserved Keratin-like coiled-coil domain (K-box), and a highly variable, often species-specific, C-terminal domain (Theißen et al., 1996; Alvarez-Buylla et al., 2000; Smaczniak et al., 2012). Type II *MADS-box* genes are also known as *MIKC* genes and can be further subdivided into *MIKC* ^c^ and MIKC* types (Henschel et al., 2002). *MIKC* ^c^ genes comprise several sub-groups including the well-known ABC(D)E genes crucial for floral organ determination and development (Becker and Theißen, 2003; Theißen et al., 2016). Research results on Asteraceae floral development particularly come from the classical model *Gerbera* (Mutisioideae; Zhang et al., 2017) and more recently from crops such as lettuce (Cichorioideae; Ning et al., 2019), sunflower (Asteroideae; Dezar, 2003), and chrysanthemum (Asteroideae; Won et al., 2021). For example, in *Gerbera*, a total of eight *SEPALLATA*-like (*SEP*-like; class E) genes were found (Zhang et al., 2017), whereas *Arabidopsis* has only four *SEP*-like genes. Unlike the redundancy of *SEP* copies in *Arabidopsis*, the different *SEP*-like genes in *Gerbera* show sub-functionalization in floral organ development and neo-functionalization in the inflorescence meristem in addition to conserved functions (Elomaa et al., 2018). Genome-wide analysis of *MADS-box* genes in *Chrysanthemum* and lettuce identified a putative *Asteraceae-specific MADS-box* (AS-MADS) clade, genes of which the evolution and function are still unclear (Won et al., 2021).

All *TCP* genes contain a highly conserved *basic HELIX LOOP HELIX* (*bHLH*) domain by which they are divided into Class I (P) and Class II (C) (Kosugi and Ohashi, 1997; Navaud et al., 2007; Li, 2015). Class I *TCP* genes represent the *PROLIFERATING CELL FACTOR* (*PCF*) genes, while class II *TCP* genes are divided into the ubiquitous *CINCINNATA* (*CIN*) genes and angiosperm-specific *CYCLOIDEA/TEOSINTE BRANCHED1* (*CYC/TB*1) genes (Luo et al., 1996; Doebley et al., 1997; Nath et al., 2003; Martín-Trillo and Cubas, 2010). Among them, *CYC/TB1* genes are closely associated with the regulation of flower symmetry (e.g., in *Antirrhinum majus*; Luo et al., 1996). Studies of *Senecio* (Asteraceae) showed that the *CYC*2-like genes *RAY*1 and *RAY*2 are involved in the development of ray florets (Kim et al., 2008). *CYC*2 homologs control various roles in the formation of ray and disc florets in distinct Asteraceae lineages, suggesting neo-functionalization (Elomaa et al., 2018), and an extensive study found that the developmental program of making a ray flower involves functionally divergent *CYC*2-like genes in different lineages (Chen et al., 2018). However, the function of *CYC* in the formation of ligulate florets is yet unconfirmed. An understudied group of *TCP* genes is *PCF* genes (Kosugi and Ohashi, 1997), which participate in a wide range of plant growth, including flower development. The increasing number of sequenced genomes presents us with an opportunity to conduct a systematic analysis of these important *MADS-box* and *TCP* gene families in a wide range of Asteraceae species.

To study the evolution of *MADS-box* and *TCP* gene families in Asteraceae, a plant family-based phylogenomic analysis is required to gain more knowledge about the history of gene retention after Asteraceae radiation-related WGDs. Moreover, the patterns of gene movement (transpositions) could help identify potential sources of regulatory novelty induced by genomic context change. Thus, a broad range of genomic comparisons, like synteny network analysis (Zhao and Schranz, 2017), is valuable to conduct alongside phylogenetic analysis. Synteny can help determine the orthologous relationships of duplicated genes among species after complex WGDs and identify other genomic positional changes, like ancient tandem duplications and gene transpositions (Dewey, 2011; Zhao et al., 2017).

**Figure 1:**
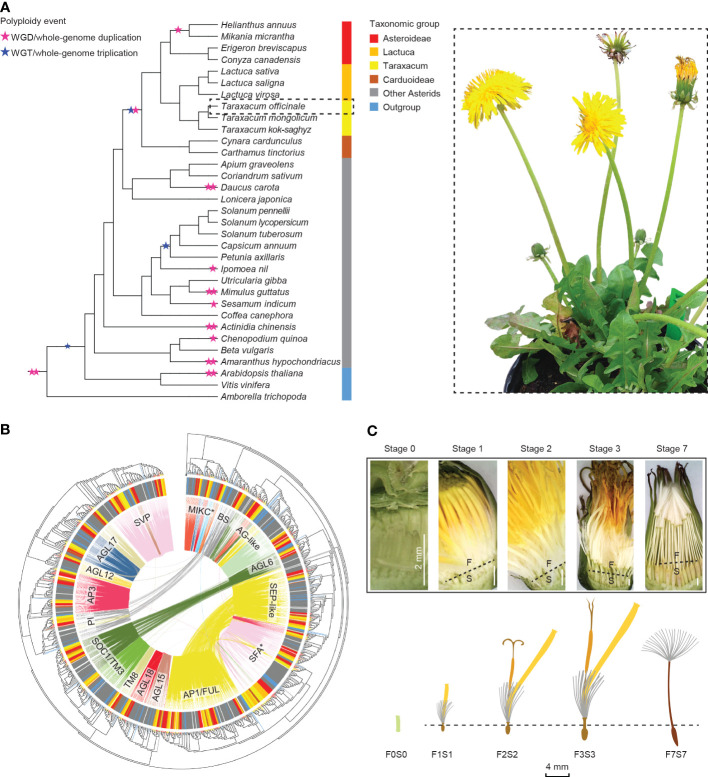
Overview of this study. **(A)** Summarized phylogeny of the angiosperms analyzed based on amino acid sequences of selected Type II *MADS-box* genes, with a focus on the Asteraceae and the position of *Taraxacum* therein and with the ancestral whole-genome duplications and triplications (stars) and subfamilies (colors) indicated (left) and a sexual dandelion plant (right). **(B)** Phylogenetic tree of the Type II *MADS-box* genes found in the species studied (see panel A), based on the MADS- and K-box domain protein sequences, with the species subfamilies in the outer circle (see panel **A** for colors), the syntenic relationships by colored lines within the circle, and the *MADS-box* gene families indicated. **(C)** Dandelion floral tissues and stages used in the gene expression analysis: F = upper floral part and S = lower floral part, separated through the beak (dotted line) except for the youngest stage (F0S0), with stage 1 = bud just before opening; 2 = open flower; 3 = 3 days after pollination (DAP); 7 = 7 DAP. ^©^ Kitty Vijverberg and Wei Xiong.

In this study, we used the common dandelion (*Taraxacum officinale*; **Figure 1**), a member of the Cichorioideae and taxonomic outgroup of lettuce, as a model. Dandelion is well-studied because of its two reproduction modes that co-occur within its distribution range: sexual diploids (2*n* = 2*x* = 16) and asexual, apomict, triploids (2*n* = 3*x* = 24) (Van Dijk et al., 1999), for example, to study the molecular genetic basis of apomixis elements including diplospory (Vijverberg et al., 2004; Vijverberg et al., 2010) and parthenogenesis (Vijverberg et al., 2019; Van Dijk et al., 2020; Underwood et al., 2022). Dandelion has been investigated for its ecological evolution and adaption (Brock et al., 2005; Verhoeven et al., 2018) and more recently for its aforementioned floret and fruit characteristics (Vijverberg et al., 2021). A genome assembly of this interesting model species will provide insights into its gene and genome evolution and serve as an important reference for comparative analysis within the Asteraceae, other *Taraxacum* species and genotypes, and related species such as lettuce, and for gene analysis and gene editing purposes.

Here, we analyzed our *de novo* assembly of a diploid sexual *T. officinale* genome together with the published whole-genome sequences of 32 other plant species. We performed genome-wide searches for *MADS-box* and *TCP* genes of the 33 species (**Figure 1A**) and constructed a synteny network of the identified genes to reveal their lineage-specific genome context and ancient tandem duplications, with a focus on the Asteraceae, its subclades Asteroideae and Cichorioideae, and *Taraxacum*. We examined the synteny versus phylogenetic trees based on *MADS-box* (**Figure 1B**) and *TCP* domain sequences and assessed a possible change in function after gene duplication or genomic context change *via* comparison to gene expression data in different floral developmental stages and tissues (**Figure 1C**) in dandelion. We also applied phylogenomic data to characterize the evolution of *Asteraceae-specific MADS* (*AS-MADS*) genes and their expression during floral development. Our results provide insights into the evolution of Asteraceae and their *MADS-box* and *TCP* genes, while the wealth of genome and transcriptome data serves as a reference for future comparative analyses and research on floral development in dandelion and beyond.

## 2 Results

### 2.1 Genome sequencing and assembly

The *T. officinale* genome of the sexual diploid plant FCh72 was sequenced with PacBio RSII and 10X Genomics on Illumina HiSeq2500 and optically mapped with BioNano. We obtained ~75× coverage of PacBio reads with a mean subread length of 12,259 bp. The reads were assembled using Canu v1.3 (Koren et al., 2017). The assembly was scaffolded with the 10X and BioNano data and polished with the 10X Illumina reads. Haplo-contigs were collapsed where possible, and the assembly was polished and scaffolded multiple times in subsequent rounds (see Materials and Methods). The resulting assembly has a total genome size of 936 Mb (**Table 1**; **Supplementary Table S1**), which is slightly larger than the expected 831 Mb based on C-values (cvalues.science.kew.org/) and significantly larger than the estimated genome size based on k-mer analysis (~614 Mb; **Supplementary Figure S1**). Blobtools confirmed the absence of contamination (**Supplementary Figure S2**). This draft genome assembly has 4,059 scaffolds, an N50 size of 757 kb, and the longest scaffold of ~23 Mb (**Supplementary Table S1**). The guanine-cytosine (GC) content is 37.0%. The mitochondrial (mt) genome was assembled in a single scaffold that showed high homology to the mt-DNA of the related species lettuce (**Supplementary Figure S3A**), whereas the chloroplast (cp) genome has not been recovered, likely due to bleaching prior to the harvesting of plant material (Material and Methods; **Supplementary Figure S3B**). Difficulties in assembling were posed by the heterozygosity of the genome, which was estimated at 1.5% with GenomeScope, showing two clear k-mer peaks (**Supplementary Figure S1**; k = 21). The Benchmarking Universal Single-Copy Orthologs (BUSCO) quality assessment of the genome assembly showed 95.4% completeness (2,219/2,326; **Table 1**), with 1,759 (75.6%) complete and single copy and 460 (19.8%) complete and duplicated genes. The high duplicated BUSCO percentage is likely due to remaining alleles, in line with partial assembly in haplo-contigs.

**Table 1:** Main characteristics of *Taraxacum officinale* genome.

Genome assembly and annotation	Statistics
Assembly size (Mb)	936
Expected genome size (Mb)	831
Number of scaffolds	4,059
N50 super-scaffolds (Kb)	757
Heterozygosity (%)	1.5
BUSCO completeness of assembly (%)	95.4
Repeats (%)	63
Predicted high confident genes	60,810
Functional annotated transcripts	56,560
Sequence identical genes (%)	2.7
Protein >99% similar genes (%)	7.5

### 2.2 Genome annotation

The assembled genome was repeat masked using RepeatModeler with long terminal repeat (LTR) detection and using RepeatMasker. In total, 63% of all bases were masked, which is similar to the repeat content of *Taraxacum mongolicum* and *Taraxacum kok-saghyz* (Lin et al., 2022). The repeat content was to a large extent driven by LTRs, namely, *Copia* (~214 Mb, 22.9% of the genome) and *Gypsy* (~135 Mb, 14.5%) retrotransposons (**Supplementary Table S2**).

The genome was annotated using an RNAseq library based on four different tissue types of the sequenced dandelion genotype—leaf, bud, open flower, and roots—using BRAKER2 (see Materials and Methods). A total of 60,810 high-confident genes (i.e., size ≧150 amino acids [aa] or ≧50 aa with homology annotation) with 63,780 transcripts were found (**Supplementary Table S3**; **Supplementary Data S1**). The mean gene length was 2,110 bp with on average 4.7 exons and a mean total Coding Sequence (CDS) length of 971 bp (**Supplementary Table S3**). For 88.7% of the genes (56,560), the transcripts have a description, and 61.4% (37,324) are associated with at least one Gene Ontology (GO) term (**Supplementary Table S3**).

A total of 1,739 high-quality genes (2.7%) were found to have at least one identical sequence copy in the annotation, and 4,788 genes (7.5%) showed more than 99% amino acid identity with another annotated gene (**Supplementary Table S3**; indicated in **Supplementary Data S1**) and are either true duplicates, closely related family members, or alleles at different haplo-contigs. The most abundant genes showed 15 and 11 copies, representing *Histone H*4 and *GOS*9-like isoforms, respectively (**Supplementary Table S3**; **Supplementary Data S1**). BUSCO analysis of the translated transcripts showed 90% completeness with 19.4% duplicated BUSCOs.

An unfiltered gene set that includes the high confidence gene models, as well as smaller transcripts (50–150 aa) and genes without homology annotation of 81,292 genes in total with 85,093 transcripts, was used in the gene expression analyses and synteny mapping results (see below).

### 2.3 Genome comparison between *Taraxacum* spp. assemblies

The *T. officinale* genome assembly was compared to that of the recently published whole-genome sequences of two other sexual diploid *Taraxacum* species, *Taraxacum mongolica* (*Tmo*) and *T. kok-saghyz* (*Tks*) (Lin et al., 2022), showing a relatively fragmented assembly (4,059 scaffolds versus 65 in *Tmo* and 160 in *Tks*; **Supplementary Table S4**). The annotation of gene space was, however, far more complete in *T. officinale* based on the BUSCO results (90% completeness versus 69% in *Tmo* and 74% in *Tks*). The GC content of 37% was similar to the other two species, whereas the heterozygosity varied from 1% (*Tks*) to 1.5% (*Tof*). The assemblies are collinear without major structural rearrangements if compared by alignments and dot plots (**Supplementary Figure S4**).

### 2.4 Synteny network analysis: identification of *Asteraceae-specific MADS-box* and *TCP* gene synteny clusters

To compare the genomic context of genes in Asteraceae and with selected outgroups, we conducted a synteny network analysis of 33 angiosperm species with high-quality whole-genome sequences (12 Asteraceae, 18 other Asterids, 2 Rosids, and 1 early-diverging Angiosperm; **Figure 1A**; **Supplementary Table S5**). Within the Asteraceae, six species were from the Cichorioideae, including our *de novo* sequenced *T. officinale* (*Tof*), two additional *Taraxacum* species (*Tmo* and *Tks*) (Lin et al., 2022), and three *Lactuca* species. In addition, four species of the Asteroideae and two of the Carduoideae were analyzed. The synteny network database was built using the SynNet pipeline (Zhao and Schranz, 2017; Gamboa-Tuz et al., 2022) and contained 718,070 nodes (genes found in syntenic blocks) and 7,603,091 edges (connections between syntenic genes), data on which subsequent analyses were based. We further focused on the sub-networks of the *MADS-box* and *TCP* gene families.

**Figure 2:**
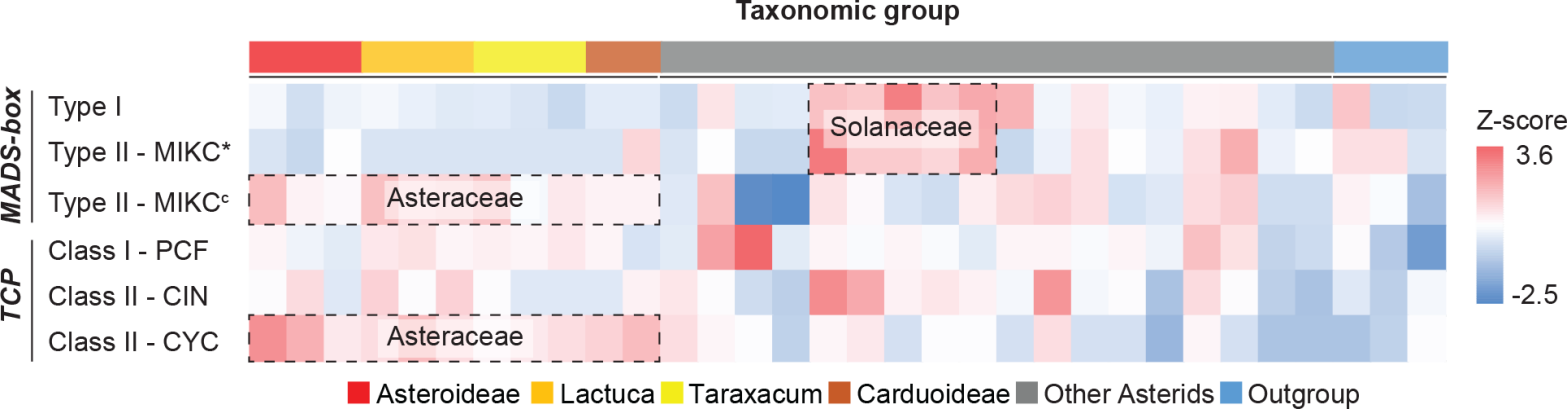
Heatmap of *MADS-box* and *TCP* gene counts normalized per gene clade. Target genes were identified and classified into sub-clades (row) for each species (column), and the count was scaled (cell) by using z-scores. Colors illustrate the deviation from average, with blue for smaller numbers and red for larger numbers. *Erigeron breviscapus* was excluded from this visualization due to its incompleteness of target genes. Species subfamilies are indicated in **Figure 1A**.

With the use of HMMER analysis of proteome sequences of the 33 species, the *MADS-box* genes were identified by searching for the *MADS-box* (SRF-TF: PF00319.20) and *K-box* (PF01486.20) domains and the *TCP* genes by searching for the *TCP*-specific *bHLH* domain (PF03634.15). We further classified the identified candidates by their sequence similarity and phylogenetic relationship to well-known reference genes, particularly from *Arabidopsis*, *Petunia*, and *Gerbera* (*MADS-box* genes), and *Arabidopsis* and rice (*TCP* genes) (**Supplementary Table S6**). In total, 2,525 *MADS-box* and 1,019 *TCP* genes were identified (**Supplementary Data S2**). After classification, the normalized gene count (i.e., Z-score) for each clade was calculated. Results identified several gene expansions in different plant families (**Figure 2**; **Supplementary Table S7**), particularly, Type I and MIKC* in the Solanaceae and *MIKC* ^c^ and *CYC* in the Asteraceae. Within *Taraxacum*, we found more *MADS-box* genes in *T. officinale* (78) than in *T. mongolicum* (54) and *T. kok-saghyz* (57) and a similar number of *TCP* genes (31-34), with the former possibly as a result of their genome completeness.

**Figure 3:**
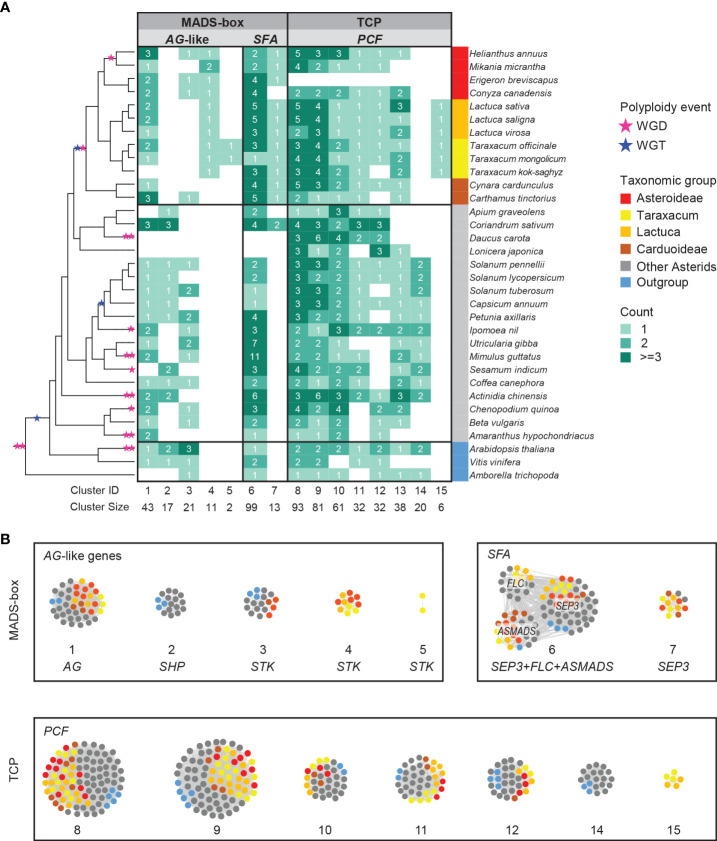
Synteny network clusters revealed the Asteraceae-specific context of several *MADS-box* and *TCP* genes important in floral development. **(A)** Phylogenetic profiling map showing a selection of *MADS-box* (*AG*-like and *SEP*, *FLC*, *AS-MADS* [SFA] clades) and *TCP* (*PCF* clade) gene clusters including the ones that showed a lineage-specific context: Cluster 4 (CL4) and CL7 for the Asteraceae, CL15 for the Cichorioideae, and CL5 for *Taraxacum*. Gradient green cells represent the number of syntelogs (syntenic homologs) for each cluster in the different species; the phylogenetic tree (left) and subspecies (right) are as in **Figure 1A**. **(B)** The same clusters are visualized in a network, particularly showing the syntenic relationships between *SEP*, *FLC*, and *AS-MADS*.

The complete lists of *MADS-box* and *TCP* genes were used to extract their synteny sub-networks from the whole network database. The resulting *MADS-box* sub-network contained 1,677 nodes and 16,697 syntenic edges, and the *TCP* sub-network contained 835 nodes and 14,716 syntenic edges (**Supplementary Data S3A, B**). To associate the syntelogs (the syntenic homologous genes) with each other, we conducted phylogenetic profiling of all obtained synteny clusters of *MADS-box* and *TCP* proteins and visualized the primary clusters in a heatmap for each family (**Supplementary Data S3C, D**). For this, the number of syntelogs in each cluster was counted for each species, and the clusters were ordered by hierarchical clustering based on the index of dissimilarity derived from the syntelog counts. Then, the clusters that were specific to the Asteraceae, Cichorioideae, and/or *Taraxacum* were determined. In **Figure 3**, we highlight 15 synteny clusters that illustrate our most relevant findings: the Asteraceae or *Taraxacum*-specific *MADS-box* clusters *AG*-like (CL4–5) and *SEP3*/*FLC*/*AS-MADS* (*SFA*; CL7), and *TCP*-*PCF* cluster 15 (CL15), and absence of *AG-*like cluster 2 (CL2) and *TCP*-*PCF* cluster 14 (CL14). The selected clusters are also displayed in a network format, pruning the non-primary syntelogs (**Figure 3B**).

The *AG*-like genes include the C class gene *AGAMOUS* (*AG*; CL1, **Figure 3**) and C/D class genes *SHATTERPROOF*-like (*SHP*-like; CL2) and *SEEDSTICK*-like (*STK*-like; CL3–5). *AG* is critical for anther and carpel development; *SHP* regulates aspects of fruit development in core eudicots, such as fruit dehiscence in dry fruits (e.g., *Arabidopsis*) and fruit expansion and ripening in fleshy fruits (e.g., tomato); *STK* is involved in ovule development. For *AG*, most orthologous genes resided in the conserved synteny cluster 1 (CL1; **Figure 3**), including two genes in *T. officinale*. Syntelog(s) of *SHP* (CL2) were absent in the Asteraceae. Since Asteraceae fruits are single-seeded indehiscent dry fruits (cypsela), this is consistent with a loss or absence of a gain of *SHP* homologs. More than 60% of the *STK* orthologs were in one single synteny cluster (CL3), mainly from non-Asteraceae species. *STK* orthologs from Asteraceae exclusively formed a second synteny cluster (CL4). Moreover, there was an extra pair of syntenic *STK* genes unique in *Taraxacum* species (CL5) and one more present in *T. officinale* only (To_STK_UN; since unique, this is not detected as a cluster). Possibly, these Asteraceae- and *Taraxacum*-specific clusters are associated with a diverged ovule developmental program in these taxa, but this needs additional support and validation.

The *SEP*3-like genes, E class genes of floral development, exemplify another Asteraceae-specific relationship. *SEP* genes underlie the development of all floral organs in many species. A conserved cluster of *SEP*3-like genes was shared by all genomes analyzed, including most Asterids, two Rosids, and the first-diverging angiosperm *Amborella trichopoda* (CL6; **Figure 3**). In addition, the majority of another *SEP*3-like cluster (CL7) was predominantly Asteraceae-specific (plus *Coriandrum sativum* [Apiaceae]) and likely a transposed duplicated copy preserved in the Asteraceae.

A third example of Asteraceae-specific synteny was found in *TCP* class II *PCF* genes, which are plant-specific transcription factors that play a role in cell differentiation and plant growth. For *PCF* genes, we identified a Cichorioideae-specific cluster (CL15; **Figure 3**), while a second cluster was specific for non-Asteraceae (CL14), hinting at a transposition in the ancestor of the Cichorioideae. The other six *PCF* clusters were relatively conserved in all species analyzed (CL8–13). Similarly, the *TCP* subclasses, *CIN* and *CYC* (**Supplementary Data S3D**), were conserved between the Asteraceae and non-Asteraceae.

### 2.5 Expression analysis of floral tissues and stages

To further analyze the lineage-specific synteny found in the Asteraceae as well as to obtain a global overview of the genetic basis underlying floral development in dandelion, we generated transcriptomes of a total of 25 samples of the sequenced plant FCh72. These included triplicates of very young whole buds (F0S0; initiating organs), older buds just before opening (F1 and S1), and open flowers (F2 and S2), the latter two stages with the florets separated into an upper (F; organs in all four floral whorls) and lower (S; the inner floral whorl/ovary/seed) part by cutting through the beak (**Figure 1C**; see for exact stages and the method of Vijverberg et al., 2021), and duplicates of these floral parts at 3 days after pollination (3 DAP; F3 and S3) and 7 DAP (F7, pappus only; S7, ripening seeds) and leaves (LF). RNA sequencing generated on average 33,8 million read pairs per sample, of which >99.9% was maintained after trimming and on average 98.1% mapped to the annotated *T. officinale* genome (see **Supplementary Table S8** for read and mapping statistics and **Supplementary Data S4** for expression values).

The quality of the data was checked with a principal coordinate analysis (**Supplementary Figure S5**). This showed clear clustering of the replicates per stage and tissues, with particularly tight clustering of replicates in the youngest stage (F0S0) and younger seed stages (S1 and S2). In the upper floral parts (F), some more variation was detected within and between replicates, reflecting fast changes in gene expression in these rapidly developing tissues and close successive stages. The leaf duplicates also nicely clustered together and diverged from the floral tissues.

Expression patterns were visualized in a heatmap (**Supplementary Figure S6**), which confirmed the reproducibility of the replicates and the quality of the data. Results showed clustering of similar tissues in subsequent stages, particularly of (F0S0), S1, S2 and F1, F2, and associated “expression blocks” (**Supplementary Figure S6**, blocks 1–12; **Supplementary Data S4**, columns S and T). Blocks with the highest numbers of genes were found among the youngest stages, particularly blocks 2 (F0S0, S1, and S2) and 3 (F1) and to a lesser extent 1 (F0S0) and 4 (F1 and F2) (**Supplementary Table S9A** and graph therein), indicating high transcriptional activity in young floral developmental stages. A relatively high number of genes was also found in block 10 (F3 and F7), indicating diverse gene activity in the degenerating florets. The overall expression pattern based on summed values over all 25 samples, and their classification in seven groups from “true zero” to “extremely high” expression (>10,000 TPM) (**Supplementary Data S4B**, columns AT, BT, BU-BX, and summary thereof in **Supplementary Table S9B**) showed a total of 49,102 genes expressed (60.4%; sum > 1 TPM), with a minority of genes with very high (7.4%; sum > 1,000 TPM) to extremely high (0.5%; sum > 10,000 TPM) expression. The comparison of Total Exon Reads (TERs) versus Unique Gene Reads (UGRs) showed similar expression for most genes (82.9%), but a small part (1.5%) showed significantly higher TERs, and a larger part (15.6%) showed significantly higher UGRs. The most highly expressed genes (**Supplementary Table S9C**) included four genes that each showed a summed expression of >100,000 TPM, of which three were related to anthers: *Pollen allergen Art v*1-like (2x) and *Anther-specific SF18*-like, and one hypothetical protein. Other highly expressed genes included *Elongation Factor* 1α (*EF*1α), *Histone* 3 (*H*3), *Acyl-CoA-binding protein*, and *Polyubiquitin* (additional information in **Supplementary Table S9**; **Supplementary Data S4B, C**).

Insights into the expression of genes related to floral development, including the *MADS-box* Type I and II genes and *TCP* genes (and *APETALA-*2 [*AP2*]), were based on the averaged expression value of genes per tissue type and stage (**Supplementary Data S4C**, with in columns F, G, and H the relevant genes indicated; **Supplementary Table S10** for extraction of these genes). Several genes (13 of the 78 *MADS-box* genes and 5 of the 33 *TCP* genes; 15%–17%) were represented by two alleles due to their assembly in haplo-contigs and taken together and their sum of expression used in the final analysis (indicated with a double gene name and asterisk in **Supplementary Table S10**). Gene expression is visualized in a heatmap per gene subclass (**Supplementary Figures S7A–D**).

The heatmap of the *MADS-box* Type II genes showed clustering of the young upper floral tissues (F0S0, F1, and F2), seed tissues (S1–7), and older upper floral tissues (F3 and F7) and a clear differential expression in leaves (LF) (**Supplementary Figure S7A**). Virtually all ABC(D)E genes (indicated with an A–E prefix in the gene name) were expressed in (subsets of) the floral tissues, confirming the expected expression patterns as well as the homology of the genes identified in the *Taraxacum* genome. For example, the class B gene *PISTILLATA* (*PI*) was highly expressed in young upper floral tissues only, and the class D gene *AGL*11-like was particularly expressed in the ovary and seed tissues. Most other *MADS-box* Type II genes (indicated with an M-prefix in the gene name) showed (very) low expression in the floral tissues, further confirming the important role of *MADS-box* Type II ABC(D)E genes in floral development.

Based on the expression of *MADS-box* Type I genes, the seed tissues clustered together as did the upper floral tissues (**Supplementary Figure S7B**), supporting their important role in ovule and seed development. A few genes were specifically expressed in young buds, *AGL*47 and *AGL*62, which also confirms expectations. The heatmap of *TCP* Class I and Class II gene expression showed a similar clustering of tissues as the *MADS-box* Type II genes (**Supplementary Figure S7C** versus **S7A**), also supporting their role in floral development. In particular, the *CIN* genes were highly expressed in the floral tissues in addition to some *PCF* genes, while most *CYC* genes showed (very) low expression. Examples of tissue specificities are the high expression of a *TCP*5-like gene in young buds and a *TCP*8-like gene in tissues after pollination (F3, S3, F7, and S7). Finally, the expression of the *AP2*-like homologs, an A class non-*MADS-box* transcription factor gene, is shown (**Supplementary Figure S7D**), of which some showed expression in the young buds according to their role in early floral organ ontogenesis.

### 2.6 Phylogenomic analysis of *MADS-box* and *TCP* genes, Synthesis

**Figure 4:**
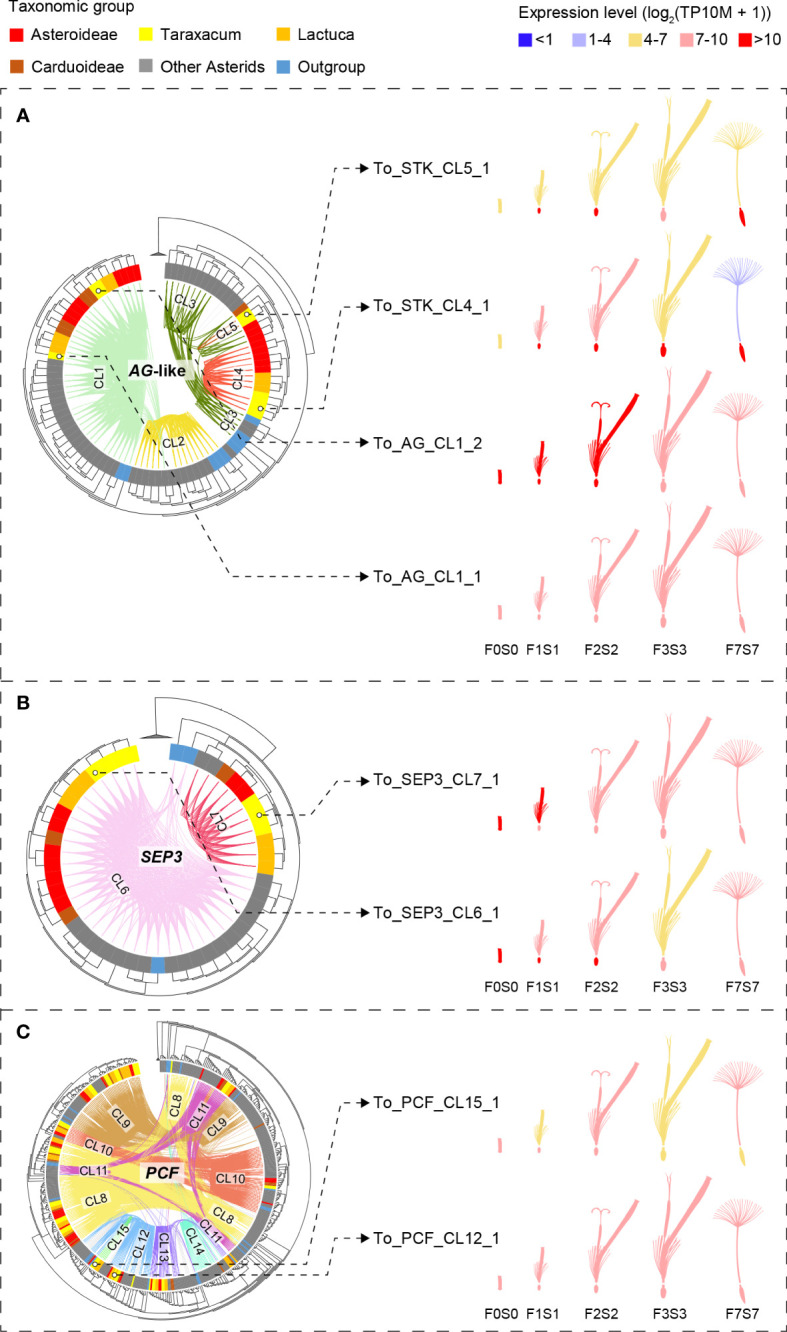
Phylogenetic trees of the *MADS-box* and *TCP* gene clades that include genes with lineage-specific genomic context (see **Figure 3**)—*AG*-like, *SEP*3, and *PCF* genes—based on *MADS-box* and *K-box* **(A, B)** and bHLH **(C)** domain amino acid alignments, respectively (left) and gene expression in different floral stages and tissues (right). Species subclades are indicated in the circle (colors as in **Figure 1A**) and synteny clusters with colored lines within the circle. **(A)** The *AG*-like clade. This shows three clades based on the gene tree and five based on their synteny: CL1 (light green) shared by all species and putatively ancestral, CL2 (the *SHP* clade, yellow), which is present only in non-Asteraceae, and CL3–5 (the *STK* clade, dark green and red lines), indicating gene duplications and divergence. Different expression patterns of the *AG*-like genes in *Taraxacum* floral tissues support their divergence. **(B)** *SEP*3 clade. This shows two clades based on both the gene sequence and syntenic relationships, indicating high conservation. CL7 is unique to the Asteraceae, possibly as a result of a transposition after gene duplication in the ancestor of the Asteraceae. Overall similar expression of the *SEP*3-like genes in *Taraxacum* supports their conservation. **(C)** *PCF* clade. This shows three subclades in the gene tree, while CL8 (yellow lines) and CL11 (pink lines) both contain syntenically connected genes from all three subclades, indicating paralogous relationships. Asteraceae-specific CL15 genes (fluorescent green lines) are monophyletic and might be a result of duplication within the second *PCF* clade (CL12 and CL13). Divergence in their expression pattern supports that these genes are diverging.

To depict the evolutionary relationships between the different *MADS-box* and *TCP* genes, we mapped the syntenic connections (genomic context) onto the gene trees (gene sequence divergence; **Figure 1B**; **Supplementary Figure S8**). Next, we extracted the subsets of genes associated with lineage specificity within the Asteraceae for more detailed analysis and comparison of their expression in dandelion (**Figure 4**). The gene trees were based on the amino acid alignments of the *MADS* domain (*MADS-box* genes) and *bHLH* domain (*TCP* genes), respectively, confirming the splitting of *MADS-box* genes into Type I and Type II (including MIKC* and *MIKC* ^c^) and *TCP* genes into *PCF*, *CIN*, and *CYC/TB1* genes. To improve the resolution of the *MADS-box MIKC* ^c^ genes, an independent phylogenetic tree was built using 1,154 Type II *MIKC* ^c^ genes where the K-box domain(s) was included in the alignment with the *MADS-box* domain (**Supplementary Data S2a, column C**; **Figures 1B**, **4A, B**). Both the phylogenies of the *MADS-box MIKC* ^c^ genes (**Figures 1B, 4A, B**) and the *TCP*-*PCF* genes (**Figure 4C**) clearly classified the various gene clades. The syntenic relationships (colored connection lines within the circles) visualize (in)congruencies with the gene evolution (phylogenetic tree), with the Asteraceae sub-families highlighted (colored sections of the circle). Both the *MIKC* ^c^ and *PCF* results showed a high level of similarity between the syntenic and gene sequence relationships, with some interesting exceptions that are described in the next paragraphs.

In **Figure 1B**, a difference between the syntenic and genetic relationships was particularly seen for *B-sister* (*BS*) versus *PISTILLATA (PI*) genes (gray lines), *AGAMOUS*-like 6 (*AGL*6) versus *SUPPRESSOR OF OVEREXPRESSION OF CONSTANS* 1 (*SOC*1)/*TOMATO MADS-box* gene 3 (*TM*3) genes (dark green lines), and *SEP*-like versus *APETALA* 1 (*AP*1)/FRUITFUL (*FUL*) genes (yellow lines). These genes show a clear close relationship based on their genomic context (are syntenic) but occur in different clades in the phylogenetic tree based on their sequences. It suggests that these genes have diverged, possibly as a result of selection or by a duplication followed by a loss of one of the two copies. **Figure 1B** also visualizes the *MADS-box* lineages that are Asteraceae specific, one within the *AG*-like clade (*STK*-like genes, red lines) and one within the *SFA* clade (*SEP*3-like genes, dark pink lines). These two syntenic clusters are shown separately in **Figures 4A, B** and described below.

The *AG*-like (*AG*, *SHP*, and *STK*) gene tree, supported by syntenic connections (**Figure 4A**, phylogeny), showed the three gene clades with five synteny clusters: the *AG* clade (CL1), shared by all species and putatively ancestral, the *SHP* clade (CL2), present in non-Asteraceae species only, and the *STK* clade (CL3–5), showing evidence for gene duplications and divergence. The separation of the three *AG*-like gene clusters is in line with the previous C/D class gene classification in angiosperms (Kramer et al., 2004). The tree validates the overall high conservation of *AG* genes and the absence of *SHP*-like genes in the Asteraceae, as mentioned above in relation to the synteny analysis (**Figure 3**). In addition, the *AG*-like gene tree shows that the genes in the *STK*-like clusters (CL3, dark green lines; CL4 and CL5, red lines) underwent different modes of evolution: the genes in CL3 are syntenically related but distributed over different clades based on their sequences, while within the Asteraceae, the genes in CL3 are genetically related to those in CL4 but syntenically diverged. The extra *STK*-like copy in *Taraxacum* (CL5), and another copy found in *T. officinale* only (To_STK_UN), suggest a unique evolution of *STK*-like genes in dandelions. Different expression patterns in the *AG*-like genes in *Taraxacum* (**Figure 4A**, floret cartoon), with overall high expression of CL1 genes (*AG*, C class, dark red), and various, less high expression of CL4, CL5, and To_STK_UN (i.e., similar to CL5, but with less high [yellow] expression in S1–S7; **Supplementary Table S10**) genes, support their divergence.

For the *SEP*3 orthologous group (**Figure 4B**, phylogeny), most gene copies resided in CL6 (light pink lines), and these form one clade in the gene tree, supporting the high conservation of this expanded gene group in genomic as well as sequence context. A second group of syntelogs was found in the Asteraceae (CL7; red lines) and is also supported by the gene tree. Possibly, these genes result from a transposition after duplication in the ancestor of the Asteraceae. The expression of the *SEP*3-like genes in *Taraxacum* (**Figure 4B**, floret cartoon) shows some reduction in mature floral tissues in the conserved, putatively ancestral, CL6 gene, and overall high expression in the Asteraceae-associated CL7 gene. This supports the importance of E class genes in a wide range of floral developmental aspects and indicates that the two gene copies have not (yet) much diverged and may have remained a similar function.

*PCF* genes form a large clade within the *TCP* genes and were divided into three subclades based on their gene tree (**Figure 4C**, phylogeny). Cluster 8 (yellow lines) and CL11 (pink lines) both contain syntenically connected genes from all three subclades, indicating their paralogous relationships. Asteraceae-specific CL15 genes (fluorescent green lines) are monophyletic according to the gene tree and likely a result of duplication within the second *PCF* clade (CL12 and CL13). Divergence in their expression pattern (**Figure 4C**, floret cartoon) suggests that these genes are diverging.

To summarize, the extracted gene trees of *AG*-like (CL1–5), *SEP*3 (CL6–7), and *PCF* genes (CL8–15) confirmed the orthologous relationship of genes within Asteraceae-specific synteny clusters (CL4, 5, 7, and 15) as well as of genes widely conserved within the angiosperms (CL1, 6, and 8–13) or being non-Asteraceae specific (e.g., CL2 and 14). By combining the gene phylogeny and synteny, we validated the occurrence of duplications and/or transpositions of *AG*-like, *SEP*3, and *PCF* genes in ancestral species of the Asteraceae or subsets thereof and added an extra level of evolutionary history to the traditional gene tree phylogenies. The comparison of the expression of these genes in different floral tissues in dandelion added another level of information by either supporting or not their divergence. Confirmation of the results by expression data in other Asteraceae in the literature, e.g., Ning et al., 2019 (lettuce) and Won et al., 2021 (*Chrysanthemum*), was inconclusive mainly as a result of different tissues and stages.

### 2.7 Presence of *AS-MADS* orthologs outside the Asteraceae and interference with their evolution, function, and relationship to *SEP*3 and *FLC*

**Figure 5:**
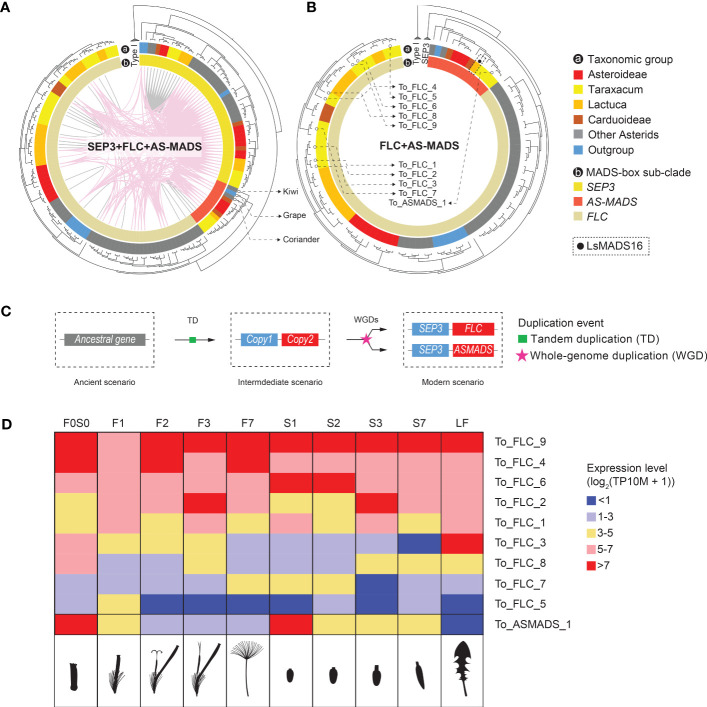
Inferred evolution of *Asteraceae-specific MADS-box* (*AS-MADS*) gene. **(A)** Phylogenetic tree of the *MADS-box SFA* clade (see **Figure 3**), based on *MADS-box* and *K-box* protein sequences, with species subclades indicated in the outer circle (A; colors as in **Figure 1A**); *SEP*3 (see also **Figure 4B**), *FLC*, and *AS-MADS* genes in the inner circle; synteny clusters with colored lines within the circle, CL6 (pink, see also **Figure 3**) and other clusters (gray); and the tree rooted by Type I MADS-box reference genes. The comparison suggests a duplication event of *AS-MADS* related to its syntelogs *SEP3* and *FLC*. The presence of *AS-MADS* (red in the inner circle) in tree non-Asteraceae species (gray and blue in the outer circle: coriander, grape, and kiwi) indicates an ancient origin of *AS-MADS* gene for all flowering plants. **(B)** Gene tree with the *SEP*3 clade excluded for a better view of the paralogous relationship between *AS-MADS* and *FLC*. The solid black dot squared by dashline points to *LsMADS*16, *AS-MADS* gene found in lettuce (Won et al., 2021). The hollow dots pinpoint the nine *FLC* and one *AS-MADS* in dandelion, for which the expression is shown in panel **(D). (C)** Hypothesized evolutionary history of *AS-MADS* in which an ancestral (Type II *MIKC* ^c^ *MADS-box*) gene underwent a tandem duplicated (TD) and then went through a polyploidy event followed by the divergence of the second copy into *FLC* and *AS-MADS*. **(D)** Expression heatmap of the nine *FLC* and one *AS-MADS* genes found in *Taraxacum officinale* in the different floral stages and tissues analyzed (indicated as cartoons below the heatmap), with the different colors indicating no or very low (blue) to medium (yellow) or high (red) expression. The results show differences between the different FLC genes and particularly a deviating expression of *AS-MADS*, suggesting neo-functionalization.

In a recent paper on *MADS-box* genes in *Chrysanthemum*, a unique, monophyletic clade was found, including 11 *Chrysanthemum* genes (*CnMADS*54–64) and one from lettuce (*LsMADS*16). These were named *Asteraceae-specific MADS-box* (*AS-MADS*) genes (Won et al., 2021). To characterize this potentially novel Asteraceae sub-group, we included *LsMADS*16 as a reference in our *MADS-box* search and annotation. Most *AS-MADS* genes were found to belong to the syntenic cluster 6 (CL6, **Figure 3**), which is one of the largest clusters in our analysis with 99 nodes. CL6 includes *SEP*3 and *FLC* in addition to *AS-MADS* genes (*SFA*), and its network showed nodes of *SEP*3-like genes that are widely but loosely connected to the sub-clusters of *FLC*-like and *AS-MADS*-like genes, both inter- and intra-specifically (**Figure 3B**, *SFA* network). We analyzed the relationships and expression of the *SFA* genes in more detail (**Figure 5**). The syntenic relationships between distant clades of the same gene family can indicate ancient tandem duplication (TD) events. For example, an ancient tandem duplication of an ancestral *MADS-box* gene in addition to several rounds of WGD is supposed to have resulted in the present tandem pairs *SEP1*-like–*AP1*, *TM3*–*AGL6*, and *SEP*3–*FLC* (**Figure 1B**). For the *SFA* cluster in our study, the synteny between *SEP*3 and *FLC* confirmed the previously identified TD (**Figure 5A**), which was further supported by the presence of a *SEP*3–*FLC* tandem of *Solanum tuberosum*, *Coffea canephora*, and *Beta vulgaris*, found in a genome-wide search for tandem duplicates of *MADS-box* genes (**Supplementary Table S11**; **Supplementary Data S5**). Moreover, syntenic relationships (i.e., colored lines connect genes in the phylogenic tree) were found between *SEP*3 and *AS-MADS* genes (**Figures 3B** [SFA plot] and **5A**), and an example of a *SEP*3–*AS-MADS* tandem was found in *Chenopodium quinoa* (**Supplementary Table S11**). Phylogenetically, the gene tree showed that *AS-MADS* is a sister clade of *FLC* (indicated in orange and beige, respectively, in the inner circle of **Figures 5A, B**), with a similar genomic context (pink connection lines within the phylogenetic tree in **Figure 5A**), indicating an extra round of duplication before the divergence of these genes after the ancient TD. In addition to WGDs, a tandem homolog of *AS-MADS*-*FLC* was found in the potato genome (**Supplementary Table S11**); therefore, a TD could also have been responsible for the paralogous clades of *AS-MADS* and *FLC*.

Based on the presence and sequence similarity of the MADS-domain, 57 potential *AS-MADS* candidates were first identified in the 33 species studied (**Supplementary Data S2A**). After filtering on the presence of also the K-box domain and curation by phylogeny, 13 *AS-MADS* genes were retained for downstream investigation (**Figures 5A, B**). Interestingly, out of these 13 genes, three were from non-Asteraceae species (**Supplementary Data S2A**), namely, *C. sativum* (coriander), *Actinidia chinensis* (kiwi), and *Vitis vinifera* (grape), whereas an *AS-MADS* gene was absent from the basal angiosperm *A. trichopoda*. This suggests that *AS-MADS* originated after the formation of the core angiosperms, but from a much more ancient root before the emergence of the Asteraceae family. Combining the synteny and phylogeny results, we infer that the *AS-MADS* and *FLC* genes probably derived from a duplication (WGD or TD) of the ancestral TD of their common ancestor (**Figure 5C**, copy 2) and the ancestor of *SEP3* (**Figure 5C**, copy 1) followed by their divergence.

As mentioned above, the *FLC* clade is close to the *AS-MADS* clade in different aspects. In lettuce and *Chrysanthemum*, the copies of *FLC* and *AS-MADS* show various expression patterns in different floral developmental stages and tissues (Ning et al., 2019; Won et al., 2021). In our dandelion genome and based on the criterion of encoding the complete *MIKC* ^c^ protein (i.e., containing the MADS domain and K-box domain), we found a total of nine *FLC* (To_FLC_1–9) and one *AS-MADS* (To_ASMADS_1) genes (indicated in **Figure 5B** and **Supplementary Table S10**, column G). These 10 genes are presented in the expression heatmap of *FLC*-like and *AS-MADS*-like genes in *T. officinale* (**Figure 5D**). All of them were expressed in at least one stage and tissue. Genes with a rather high expression (>5 with pink or red color in **Figure 5D**) can be divided into two major groups: one that shows a more general high expression, such as To_FLC_4, To_FLC_6 and To_FLC_9, and another that shows expression in a specific stage or tissue, such as To_FLC_2 in stage 3 (F3 and S3) and To_ASMADS_1 in the young bud/ovary (F0S0 and S1). Thus, the expression pattern validated the distinctiveness of To_ASMADS_1 in contrast to the different *FLC* genes in dandelion. To conclude, our phylogenomic and transcriptomic data suggest a non-Asteraceae-specific origin of *AS-MADS* genes and advocate its different function for dandelion floral development compared to *FLC*.

## 3 Discussion

Here, we provide the first complete overview and first inventory in *Taraxacum* of *MADS-box* and *TCP* genes in the Asteraceae by comparing their results with other Asterids and representative outgroups. Our search was robust, and the identified gene numbers in the selected species were comparable to those in previous studies; for example, 82 *MADS-box* genes, including 23 Type I and 59 Type II genes, were reported in lettuce (Ning et al., 2019), while we found 78, including 20 Type I and 58 Type II *MADS-box* genes (**Supplementary Table S7**). A comparative analysis of the *TCP* genes in the Apiaceae family identified 29 genes in *Apium graveolens*, 43 in *C. sativum*, and 50 in *Daucus carota* (Pei et al., 2021), while we found 32, 45, and 50 members, respectively (**Supplementary Table S7**). In line with this, we found 27–41 *TCP* genes in the Asteraceae (with one exception of five genes in *Erigeron breviscapus*) and 31–34 in the three *Taraxacum* species.

### 3.1 Unique patterns of gene family expansion and loss of *MIKC* ^c^ *MADS-box* and *CYC/TB1 TCP* genes in Asteraceae

After classification of the identified *MADS-box* genes, we found that the Asteraceae, represented by 12 species in our analysis, contained a lower number of Type I (on average 18.5) and Type II MIKC* (on average 2.8) *MADS-box* genes as compared to the other selected eudicots (on average 41.8 Type I and 6.1 MIKC* genes; **Supplementary Table S7**). In comparison to the Asteraceae, the Solanaceae, represented by five species in our study, have around four times as many Type I (on average 74.8) and Type II MIKC* (on average 11.0) *MADS-box* genes, which indicates a larger gene retention after its recent whole-genome triplication (WGT) (91–52 Mya; Sato et al., 2012). Instead, the Asteraceae underwent a *MADS-box* Type II gene family expansion and retention after a WGT (from two successive rounds of paleopolyploidy; Barker et al., 2008; Barker et al., 2016), retaining more *MIKC* ^c^ genes (on average 45.8) than the other eudicots (on average 36.4), such as in the Solanaceae (on average 37.2; **Supplementary Table S7**). Thus, both the Solanaceae and Asteraceae show lineage-specific gene expansions and high levels of gene retention of Type I/Type II MIKC* and Type II *MIKC* ^c^ *MADS-box* genes following a WGT, respectively (**Figure 2**). These multiplicated copies might evolve into new functions; for example, in *Gerbera*, eight *SEP*-like *GERBERA REGULATOR OF CAPITULUM DEVELOPMENT* (*GRCD*) genes are present that individually show conserved, sub-functional, and neo-functional roles in floral organ development, in contrast to the four redundant copies in *Arabidopsis* (reviewed by Elomaa et al., 2018). Moreover, diverged expression patterns of the duplicated genes found in our study suggest a possible novelty in regulation or function (**Figures 3**–**5** and below).

A similar scenario of gene expansion was found for *TCP* Class II *CYC* genes (**Figure 2**), where the Asteraceae (except for *E. breviscapus*) contained nearly twice as many *CYC* genes (on average 10.7) as compared to the non-Asteraceae (on average 5.9; **Supplementary Table S7**). This extensive *CYC* duplication has been reported for many specific Asteraceae (sub)families (Chapman et al., 2008; Kim et al., 2008; Tahtiharju et al., 2012; Huang et al., 2016; Chen et al., 2018), including Senecioneae (*Senecio*), Mutisieae (*Gerbera*), Asteroideae/Heliantheae (sunflower), and Asteroideae/Anthemideae (*Chrysanthemum*). Our study further supports the duplication of *CYC* genes in the Cichorioideae, represented by dandelion and lettuce, and Carduoideae, represented by cardoon and safflower, reiterating a whole-family duplication event of the *CYC* clade. Moreover, the regulatory function of duplicated *CYC* genes for Asteraceae ray floret development reported for *Gerbera* and sunflower can be assigned to different copies in the *CYC*2 clade (Chapman et al., 2008; Tahtiharju et al., 2012), while the loss of one of the *CYC*2 genes (*CYC*2d) was found associated with the formation of ligulate florets (Cichorioideae; Chen et al., 2018). In our phylogeny (**Supplementary Figure S8**), we also classified the *CYC* genes based on the study from Tahtiharju et al. (2012), using reference genes of *Arabidopsis*. The classified *CYC*2 clade contains two genes from *T. officinale*, Toff_WURv1_g24074, and Toff_WURv1_g36520, and the high expression of Toff_WURv1_g36520 in the young floral tissues, before fertilization (stages 0–2), suggests a role in the regulation and development of the formation of ligulate florets in dandelion, while Toff_WURv1_g24074 was not expressed during floral development (**Supplementary Table S10**). Genes from other Asteraceae species in the *CYC*2 clade should also be checked experimentally to determine their function in floral organ identity (**Supplementary Figure S8**).

### 3.2 Lineage-specific gene loss and genomic context of *MADS-box* and *TCP* genes

Our phylogeny shows that the *AGAMOUS*-like (*AG*-like) clade of *MADS-box* genes also contains *SHATTERPROOF* (*SHP*) and *SEEDSTICK* (*STK*) (**Figure 4A**, represented by CL2 and CL3–5 respectively; see also **Figure 3A**), agreeing with their close relationship in previous phylogenies and classifications (Theissen et al., 2000). In the model plant *Arabidopsis thaliana*, *SHP* and *STK* are both involved in ovule/fruit development: *SHP* can activate an *AG*-independent carpel developmental pathway and subsequently control fruit dehiscence for seed dispersal (Liljegren et al., 2000; Pinyopich et al., 2003), and *STK* regulates the development of the funiculus to connect the seed to the ovary wall/placenta (Pinyopich et al., 2003). Interestingly, our phylogenomic results reveal that the *SHP*-type (CL2) is absent from all Asteraceae species analyzed, while it is present in most other angiosperms (**Figure 3A**), and the *STK* type of the Asteraceae (CL4; **Figure 4A**) is primarily located in a different genomic context than in the other angiosperms (CL3). In a previous phylogeny of eudicot *MADS-box* genes, the *PLENA* (*PLE*) lineage of clustered *SHP*s lacked proteins from the Asteraceae, confirming that *SHP* genes are missing in this family (Dreni and Kater, 2014). Compared to the Brassicaceae (*Arabidopsis*), the observed absence of *SHP* and presence of specific *STK* copies potentially could be linked to the Asteraceae unique mode of seed dispersal by wind or animals through the pappus (Jana and Mukherjee, 2012) rather than *via* shattering (Liljegren et al., 2000). This may suggest that diversification into *SHP* has not occurred in the Asteraceae or has become lost, although some type of dehiscence still occurs in the Asteraceae to release the cypsela from the receptacle. The *STK* transposition might have influenced the fruit differences, such as the formation of single integument ovules and single seed ovaries in Asteraceae versus the double integument ovules and multiple seed-containing ovaries in the Brassicaceae, which need further validation.

*SEP*-like (class E) genes are essential regulators that orchestrate the formation of different floral organs in combination with the other ABC(D) genes (Theißen et al., 2016). The *SEP*-like genes can be divided into the *SEP*1/2/4 clade and the *SEP*3 clade in *Arabidopsis*, where *SEP*3 has been shown to co-regulate the activation of B and C class *MADS-box* genes, being involved in all floral organs but the sepals (Pelaz et al., 2000; Rijpkema et al., 2010). In *Gerbera*, eight *SEP*-like genes are found, the GRCD genes of which *GRCD*4 and *GRCD*5 co-regulate with the B class genes and *GRCD*1 and *GRCD*2 with the C class genes in petal end stamen and carpel formation, respectively, showing an expansion and sub-functionalization of the *SEP*3 function in this Asteraceae species (Zhang et al., 2017). We found that also in other Asteraceae (represented by 12 species of three subfamilies), gene duplication of *SEP*3 lineage genes is present and revealed one Asteraceae-dominant clade of *SEP*3 genes with lineage-specific synteny (CL7) compared to a conserved synteny shared by the other angiosperms (CL6; **Figures 3**, **4B**, **5A**). *T. officinale* has two *SEP*3 genes with a complete Type II structure, one in each cluster (To_SEP3_CL6_1 and To_SEP3_CL7_1 in **Figure 4B**). The similar expression of the two *SEP*3-like genes in *T. officinale* does not hint at a divergence in function; however, considering the diverse functions of *SEP*3-like genes in *Gerbera* (reviewed by Elomaa et al., 2018), it will be interesting to examine patterns of neo- and sub-functionalization of different *SEP*3 copies also in *T. officinale*, as well as the potential effect caused by a positional change (i.e., their different syntenies).

Although *CYC* genes are the typical *TCP* genes of interest for their control of flower symmetry (Luo et al., 1996), we also included the other class of *TCP* genes in our analysis, *PCF* genes, and we found an Asteraceae-specific synteny of these genes (**Figures 3**, **4C**, CL15). Overall, we found a syntenic depth of three for *PCF* genes, which is likely derived and retained from the ancient γ WGT shared by the eudicots. Compared to the function of the *CYC* genes, *PCF* genes (*PCF*1 and *PCF*2) were first defined in rice and found to regulate the expression of meristematic tissues primarily *via* heterodimers (Kosugi and Ohashi, 1997). Finding an Asteraceae-specific synteny of *PCF* genes might, therefore, imply that they evolved a unique role in aspects of Asteraceae meristem development. The different expression of the two *PCF* genes in *T. officinale*, To_PCF_CL12_1 and To_PCF_CL15_1 (cartoon in **Figure 4C**), implies a functional or regulatory novelty. Further analysis of these genes and a test of their *cis*-regulatory elements to examine whether PCF_CL15 has evolved a regulatory novelty under the Asteraceae-specific genomic context could shed more light on this specific finding.

### 3.3 Origin and revised classification of *AS-MADS* gene

*Asteraceae-specific MADS-box* gene (*AS-MADS*) was recently described by Won et al. (2021), who identified a monophyletic clade comprising multiple *AS-MADS* copies from *Chrysanthemum* and one copy from lettuce. The single lettuce copy (*LsMADS*16) was earlier found to be in the *FLC*-like clade (Ning et al., 2019). In this study, we identified a monophyletic clade of *AS-MADS* genes anchored by *LsMADS*16 (**Figure 5B**, indicated with a black dot). Surprisingly, the *AS-MADS* clade also contains genes from coriander and kiwi (Apiaceae as Asteraceae outgroup and basal Asterid; both indicated in **Figure 5C** phylogenetic tree) and grape (basal rosid) in addition to those in the Asteraceae. Our phylogenomic analysis also demonstrated that *AS-MADS* and *FLC* share the same common ancestor and are both syntenic to *SEP*3 (**Figures 5A, B**). The previous study proved that *FLC* shares the same ancestor with *SEP* before the ancient tandem duplication event in seed plants (Ruelens et al., 2013; Gramzow et al., 2014). Based on our phylogenomic analysis, we propose that *AS-MADS* is also derived from the same ancestral gene of *SEP* and *FLC* (**Figure 5B**, visualized as copy 2). Moreover, the synteny of *AS-MADS* is maintained in one single cluster comprised of Asteraceae species and other eudicots, which indicates that this synteny has been retained for *AS-MADS* at least since the last common ancestor of the eudicots. Furthermore, *FLC* was found in a more ancestral species, *A. trichopoda* (basal angiosperm); hence, its paralog *AS-MADS* could also diverge from the ancient ζ WGD (i.e., shared by all angiosperms) after the *SEP*3–*TM*8 tandem arose in the ancient flower plant ancestor. In summary, our results anticipated that the *Asteraceae-specific MADS* is a paralog of *FLC* and has a more ancient origin than previously thought but is prevalently reserved to the Asteraceae compared to other eudicots.

### 3.4 Expression patterns of lineage-specific genes suggest specialized functions/novel regulation in floral development

By combining phylogeny and synteny information, we have validated and expanded gene family classifications and the identification of orthologous relationships between genes in conserved and lineage-specific genomic contexts (**Figures 4**, **5**). Based on phylogenomic analyses, we further examined the expression of genes that we found associated with the Asteraceae, including *STK*-like, *SEP*3-like, *PCF*-like, and *AS-MADS* genes, using our *T. officinale* genome and floral transcriptome data as references. We found a diverse pattern of expression (**Figures 4**, **5D**, and **Supplementary Figure S7**). The *STK* copy in *T. officinale*-specific synteny (To_STK_CL5_1) has a partial expression pattern of another copy in Asteraceae-specific synteny (To_STK_CL4_1), implying a potential sub-functionalization event. For *SEP*3, the expression pattern of the *T. officinale* copy in the Asteraceae-specific context is highly similar to the second copy in the conserved synteny with other angiosperms, which indicates a conserved function. Unlike *SEP*3, the two closely related *T. officinale PCF* genes showed a different expression during floral development, which indicates a potential regulatory novelty after gene transposition. In *Phalaenopsis* species, *PCF* genes were found to co-express with other transcription factors like *MADS-box* (e.g., *AP*3, *PI*, and *SEP*3) and *MYB* (e.g., *TCP*) genes in buds, callus, and gynostemium (Pramanik et al., 2020). A similar balancing role might be true for either one of the *PCF* copies. For *AS-MADS*, the one *T. officinale* copy has a different expression pattern than the other nine *FLC* copies, which is highly expressed in the young bud (F0S0 and S1). This result suggests the specialization of *AS-MADS* genes as a separate subclade of the *MIKC* ^c^ type.

### 3.5 Concluding remarks

We sequenced the genome and transcriptome of the common dandelion. While the genome assembly is fragmented as compared to genomes based on the latest long-read sequencing methods, it has good completeness in terms of both assembly and annotation. The work presented in our study shows the usefulness of the *de novo* genome and floral transcriptomes of *T. officinale* for unraveling aspects of the evolutionary history of the Asterids, Asteraceae, Cichorioideae, and/or *Taraxacum*. Combining these *de novo* genome sequences with genomic data of other Asterids, we systematically studied the two most important gene families related to floral development, the *MADS-box* and *TCP* genes, and revealed a handful of interesting, Asteraceae (sub)family-related uniqueness. Future high-quality genome assemblies of other Asteraceae species can facilitate and validate our conclusions about *MADS-box* and *TCP* contribution to Asteraceae floral evolution. We also validated gene expression in lineage-specific synteny or phylogeny (*AS-MADS*) using referenced-based mapping on our *T. officinale* genome. Validation of the gene expression in other Asteraceae by comparison to data from literature was inconclusive and needs investigation in a single study with various species for which the present study provides interesting guidelines. In addition to the different floral states, *T. officinale* material from the inflorescence meristem stages would be interesting to sequence to further explore the function of *MADS-box* and *TCP* yet highlighted in this paper.

## 4 Material and methods

### 4.1 Plant material

The common dandelion accession sequenced is a sexual diploid member of a dandelion population in France near the village of Châtillon, Jura (FCh72; population F3 in Verhoeven and Biere, 2013). It was grown from a field-collected seed and maintained in the greenhouse *via* cuttings, under 16/8-h light/dark conditions, frost-free, and a maximum temperature of 20°C. FCh72 is a plant with 2*n* = 2*x* = 16 chromosomes and an estimated genome size of 831 Mb (Doležel and Bartoš, 2005).

### 4.2 DNA preparation

One of the cuttings of plant FCh72 was placed in the dark (etiolated) for 3 days, after which young leaves were harvested, the largest veins were removed, and the remainder were frozen in liquid N_2_ and stored at −80°C. DNA extraction was performed according to the cetyltrimethylammonium bromide (CTAB) method by Chang et al. (1993) with minor modification, while care was taken in all steps to keep the high-molecular-weight (HMW) DNA. In brief, a total of 2–3-g leaf material was grounded in liquid N_2_, the DNA was extracted in pre-warmed CTAB buffer at 65°C for 1 h, and the DNA was purified *via* two subsequent chloroform extractions and then precipitated using 0.7 volumes of isopropanol (4°C overnight). Pellets were resuspended in 450 µl of RNase- and DNase-free MilliQ water (MQ), and the RNA was removed by an RNase treatment with 50 Units RNaseOne™ Ribonuclease (Promega, Madison, WI, USA). An equal volume of sodium chloride–Tris-EDTA (SSTE) 2× buffer was added, a third chloroform extraction was performed, and the DNA precipitation was in ethanol. DNA pellets were dissolved in MQ, and the concentration and quality were examined on a NanoDrop 2000 (Thermo Scientific, Waltham, MA, USA) and Qubit 2.0 (Invitrogen, Life Technologies, Carlsbad, CA, USA), with the latter using the dsDNA HS assay (Invitrogen, Life Technologies). A total of 40 µg of HMW DNA was prepared for PacBio and Illumina library preparations for sequencing.

### 4.3 RNA preparation

To facilitate gene annotation, a mix of RNA from *T. officinale* flower, bud, leaf, and root tissues was prepared. Tissues were collected from cuttings of the mentioned plant FCh72 over different days, depending on tissue availability. The largest veins were removed from the leaves, and the roots were quickly rinsed with MQ, after which the tissues were frozen in liquid N_2_ and stored at −80°C. Total RNAs were extracted from each of the tissue types separately following the TRIzol reagent (Invitrogen) method with the adjustments by Ferreira de Carvalho et al. (2016). RNAs were treated with DNAse (Turbo DNA free™ kit; Ambion, Austin, TX, USA) according to the manufacturer’s protocol. The RNA integrity and concentration were checked on a NanoDrop 2000 and by examining the 25S:18S quality and ratio on a 1% agarose gel. Samples were then pooled to equimolar concentrations, and a total of 1.5 µg of RNA was prepared for Illumina library preparation.

For floral expression analysis, RNAs from *T. officinale* flower buds and heads at different developmental stages were harvested from cuttings of plant FCh72, with the younger stages (stages 0, 1, and 2) in triplicate and the older stages (stages 3 and 7) as duplicates. Harvesting was performed over different days, depending on tissue availability. The samples included very young, whole buds (F0S0; initiating organs, stem ~0 cm; a mix of three buds) and buds and heads of older stages separated through the beaks in upper (F; organs in all four floral whorls) and lower (S; the inner floral whorl/ovary/seed) floral parts: mature buds (F1 and S1; organs determined and elongated, stem ~10 cm), open flowers (F2 and S2) and old flowers (F3 and S3; 3 days after pollination [DAP]), mature pappus (F7) and ripening seeds (S7; 7 DAP), and leaves (LF) (see for exact stage definitions and sample preparation Vijverberg et al., 2021). A total of 10–40-mg tissue was collected for each sample, quickly prepared at room temperature, and then frozen in N_2_. Total RNAs were isolated using TRIzol reagent (Invitrogen, Life Technologies) as described above and dissolved in DEPC-MQ to a final concentration of 200 ng/µl.

### 4.4 DNA and RNA sequencing

The *Taraxacum* genome was sequenced in three rounds by using a PacBio RSII sequencing system (Pacific Biosciences), 10X Genomics combined with Illumina HiSeq2500 125 paired-end sequencing (Leiden, The Netherlands), and BioNano Genomics Technology. All library preparations and sequencing were performed by the sequencing facility of Wageningen University & Research, The Netherlands. PacBio uses Single Molecule Real-Time (SMRT) sequencing technology, providing long reads averaging 10–15 kb. The 10X Genomics method is droplet-based, enabling barcode-specific sequencing of small amounts of DNAs/single DNA strands, facilitating the haplotype detection and sequence assembly. The Illumina reads were also used to polish the sequences. Optical mapping by BioNano further improved the contig assembly.

The RNA library preparations and sequencing for gene annotation were performed at the same sequence facility at Wageningen University & Research, using Illumina HiSeq2500 125-nt paired-end sequencing. The RNA library preparation and sequencing of samples of the floral expression analysis were performed at BaseClear BV (Leiden, The Netherlands), using Illumina NovaSeq 150-nt paired-end sequencing.

### 4.5 Genome assembly

We obtained PacBio reads with the mean subread length of 12,259 bp and a total length of 62,496,657,252 bp, corresponding to ~75× coverage of the *Taraxacum* genome. In addition, we obtained ~161× of Illumina 10X 150-nt paired-end reads. The PacBio subreads were assembled using Canu (version 1.3, corMaxEvidenceErate 0.15) (Koren et al., 2017). The resulting contig assembly was checked for contaminants using blobtools (v1.0) (Laetsch and Blaxter, 2017) and assessed for completeness with BUSCO (v5.2.2 using eudicot_odb10) (Manni et al., 2021). Assembly statistics were gathered using Quast (v5.02) (Gurevich et al., 2013). To collapse separately assembled haplo-contigs, the purge_dups manual protocol was followed. In brief, any contigs with assembly ambiguities were split using tigmint (v1.0.0) (Jackman et al., 2018), reads were mapped back to the split assembly using minimap2 (Li, 2018), and putative haplo-contigs were collapsed by purge_dups using coverage information. Internal joins in scaffolds by purge_dups were then split on all 22N recognition sequences. This assembly was then polished with two rounds of RACON (v1.4.11) (Vaser et al., 2017) using the original PacBio data. Next, the polished assembly was scaffolded with the Illumina 10X data using ARCS (v1.1.0) (Yeo et al., 2018). The assembly was further scaffolded with BioNano Irys data using hybrid scaffolding. In the final step, the assembly was polished with the 10X Illumina data using Pilon (v1.22) (Walker et al., 2014).

### 4.6 Repeat masking

Repetitive sequences and transposable elements (TEs) in the *T. officinale* genome were identified using a combination of *de novo* and homology-based approaches at the DNA level. *De novo*: RepeatModeler (v.2.0.1 with the LTRstruct option) was used to create a *de novo* repeat dataset (Flynn et al., 2020). The results from RepeatModeler were combined with the RepeatMasker combined data subset relevant for *T. officinale* (i.e., Viridiplantae) and used as input for RepeatMasker (open-4.0). The results from RepeatMasker were used to soft-mask the genome assembly prior to annotation (Smit et al., 2019).

### 4.7 Gene prediction and functional annotation

We employed the BRAKER2 (Brůna et al., 2021) pipeline for *ab initio* gene prediction. First, stranded RNAseq data from four tissues were quality and adapter trimmed using Cutadapt (v1.11) (Martin, 2011). The trimmed reads were aligned against the assembly (sans mitochondrial scaffold) using STAR (v2.6.1c) (Dobin et al., 2013). The aligned reads were separated into forward and reverse reads for BRAKER2 stranded mode. The reads were used as input for BRAKER2, together with the soft-masked reference. The BRAKER2 RNA evidence-based pipeline uses GeneMark-ET (Lomsadze et al., 2014) to generate initial gene structures using transcript support from RNAseq alignment. Next, AUGUSTUS (Stanke et al., 2008) uses the filtered predicted genes for parameter training and then integrates RNAseq information as extrinsic evidence into final gene predictions. For functional annotation and filtering, the transcript sequences predicted by BRAKER2 were extracted using gffread (Pertea and Pertea, 2020) and converted to protein sequences using EMBOSS transeq (v6.6.0; Rice et al., 2000). To identify homologous sequences, we used DIAMOND (Buchfink et al., 2021) blastp (v2.0.7, “-b 10 –c1 –outfmt 5 –sensitive”) against nr (downloaded 06-03-2021). In addition, we analyzed the transcripts with InterProScan (v5.50-84.0) (Jones et al., 2014). Protein sequences, blast output, and InterProScan results were then imported into Blast2Go (Conesa et al., 2005) Basic (v5.2.5) and annotated with gene names and GO terms following the standard annotation pipeline. The resulting annotation was exported as gff3 file and subsequently formatted, filtered, and annotated using custom scripts. Mainly, transcripts shorter than 150 aa without homologous evidence were removed, duplicated transcripts marked in the Note field with “Sequence identical to:” and transcripts with more than 99% aa identity were labeled with “Protein >99 perc identical to:” followed by the matching gene identifiers. Genes were relabeled in order of appearance on the assembly.

### 4.8 Genome comparison

We aligned our assembly with those of *T. mongolica* and *T. kok-saghy*z using minimap2 (v2.24-r1122: -x asm5 -K 4g –cap-kalloc=2000m -t 16) and visualized the outcome in a dot plot using Dotplotly (-s -t -m 5000 -q 50000 -k 40-x). We ran BUSCO (v5.2.2 with using eudicots_odb10) on three transcriptomes to compare genome quality (see also section below).

### 4.9 Genome database

Plant whole-genome sequences of 33 species were selected for synteny network and phylogenomic analysis, including species of the two large, derived crown groups of the Asteraceae (Cichorioideae (covering *Taraxacum*) and Asteroideae), two species of a basal subfamily (Carduoideae) four none-Asteraceae Asterid II members, 11 species from the Asterid I clade, four early-diverging Asterids, two species of the Rosids, and the basal *A. trichopoda* (**Supplementary Table S5**). Among them, 14 were retrieved from Zhao and Schranz (2019), 19 from more recent studies (mainly Asteraceae), and one *de novo* sequenced in this study (*T. officinale*). The protein sequences of primary transcripts and corresponding gene positions were extracted from selected genomes for downstream phylogenic and syntenic analyses. BUSCO (v5.2.2) was used to assess the completeness of proteomes using the eudicots_odb10 dataset.

### 4.10 Identification and classification of *MADS-box* and *TCP* genes

For *MADS-box* genes, HMMER (v3.3.2) was used to search for the *MADS-box* (PF00319.20) and *K-box* (PF01486.20) domains in all amino acid (aa) sequences of all 33 species, with a default cutoff using the profiles of hidden Markov models (HMMs) collected from pfam (Mistry et al., 2013). To classify the identified *MADS-box* candidates, a reference database of 162 *MADS-box* genes was prepared, including 107 from *A. thaliana*, 32 from *Petunia hybrida*, 21 from *Gerbera hybrida*, one from *Solanum lycopersicum* (tomato) (TM8), and one *Asteraceae-specific MADS-box* gene (*AS-MADS*) from *Lactuca sativa* (lettuce) (**Supplementary Table S6**). To quickly classify the sub-families of the identified *MADS-box* genes, BLAST (v2.12.0) was applied to search for the best match of each candidate using aa sequence encoded by the reference genes as the database with default cutoff.

For *TCP* genes, 53 classified genes were collected, including 24 from *A. thaliana*, 26 from *Oryza sativa* (rice), two from *A. majus* (Garden snapdragon), and one from *Zea mays* (maize). The source of the sequence data from this section can be found in **Supplementary Table S6**. HMMER was used to search for the *TCP* domain (PF03634.15) in 33 proteomes with the default setting. To further classify the *TCP* homologs, BLAST (v2.12.0) was applied to search for the best match for each candidate using the reference genes as a database.

### 4.11 Synteny network analysis

Complete synteny networks of proteomes for the 33 plant species were created by the SynNet-Pipeline from (Zhao and Schranz, 2017; https://github.com/zhaotao1987/SynNet-Pipeline). In this pipeline, Diamond (v2) was applied to conduct the whole-genome protein comparison (Buchfink et al., 2015). Then, MCScanX was used to detect the syntenic blocks (minimum homologs = 6 genes, max gaps =25 genes), and the output was merged into the synteny network database (Wang et al., 2012). The syntenic connections of identified *MADS-box* and *TCP* genes were extracted from the synteny network (**Supplementary Data S2**). Then, extracted sub-networks were further clustered (i.e., cut into small networks) by the Infomap algorithm in R (Rosvall and Bergstrom, 2008). Clustered synteny networks were visualized in CYTOSCAPE (v3.7.1) (Shannon et al., 2003). Next, phylogenomic profiles were built by quantifying syntenic genes per syntelog (syntenic homolog) cluster in all 33 species. Subsequently, hierarchical clustering (ward.D) was performed to re-order the synteny clusters using the Jaccard index. To study the genomic context of interesting genes, clusters were annotated by their primary syntelog(s) (>10% composition). Clusters were determined as Asteraceae-specific if more than 80% of the syntelogs were from Asteraceae species. The 80% cutoff instead of 100% was selected to maintain the evidence of closely related species that shared the same WGD or WTD events with the Asteraceae.

### 4.12 Expression analysis

Analysis of gene expression and visualization thereof was performed by using CLC-Genomic Workbench (CLC-GW, v20; Qiagen), Excel, and R (v4.0; The R Foundation). For 13 *MADS-box* and 5 *TCP* genes, they were represented by two alleles (i.e., due to assembly in two haplo-contigs), identified by sequence similarity, partiality of some gene(s), flanking sequences, and expression pattern. The maximum distance between paired reads was set to 2,000 nt; raw sequence reads were trimmed on quality (0.05), ambiguity (2nt), adapters, and length (>30 nt); and both paired and broken pairs were saved for mapping. Samples with high read numbers were sampled back to 60 M single reads by using the “Random sampling” tool. Read mapping was performed to the annotated *Taraxacum* genome, including all genes of length 150 nt and longer, using the “RNAseq analysis” tool and the following settings: Mismatch cost = 2, Insertion cost = 3, Deletion cost = 3, Length fraction = 0.5, and Similarity fraction = 0.9. Three expression values were collected: TERs, UGRs, and Unique Exon Reads (UERs). For measuring the UGRs, all genes were extended with an extra 400 nt up- and downstream of the genes to ensure including the reads that map partly or entirely in the 5′- and 3′-UTRs in the counts. For the final analysis and heatmaps, Total Exon Reads were used after normalization to Transcripts Per Million (TPM) in CLC-GW. The data were checked for quality using principal component analysis (PCA) and a heatmap, using the “PCA for RNAseq” and “Create heatmap for RNAseq” tools, respectively, with the latter based on Euclidean distances and complete cluster linkage. In the heatmap, stage(s)- and tissue(s)-related “expression blocks” were defined manually, and the corresponding “block” numbers were added to the genes involved. The data were then exported to Excel, and the overall expression pattern was analyzed by summing the TERs and UGRs (in TPM) over all 25 samples for each gene. Summed expressions were classified into seven groups from “true zero” to “extremely high” expression (>10,000 TPM), and ratios were compared, with higher TERs explained by the total versus unique mapping of reads and higher UGRs by mapping of reads to introns and 400-nt untranslated regions (UTRs) in addition to the exons. Read values were then averaged over replicates. For this, the raw values were transformed to reads per ten million (RP10M) and averaged (AvTERs), and then the averaged values were transformed to TP10M. Subsequently, the Minimum (MIN), Maximum (MAX), Range (MAX-MIN), and Ratio (MAX/MIN) were calculated over the nine floral-related tissue types, excluding the leaf sample, for each gene. In the cases in which MIN = 0, the ratio was based on the lowest non-0 value and the number of samples without expression indicated. Heatmaps of subsets of genes were calculated based on the averaged values after excluding all non-expressed genes (MAX < 5) transformation to Log2(AvTER +1), using Heatmaply in R, with the subsets of genes of interest: *MADS-box* genes and *TCP* genes.

### 4.13 Phylogeny reconstruction of identified genes

For both *MADS-box* and *TCP* genes, the protein sequences of all identified homologs were aligned based on their domains’ HMM (PF00319.20 and PF03634.15) using HmmerAlign (Kristensen et al., 2011). Next, PAL2NAL (v14) was used to convert the protein alignments back to codon alignment, and the codon alignments of *MADS-box* and *TCP* genes were trimmed by TrimAl (v1.4.1) using -automated1 and -gappyout mode, respectively (Suyama et al., 2006; Capella-Gutiérrez et al., 2009). RAxML phylogenetic trees were constructed for both gene families by IQ-TREE (v1.6.2) with 1,000 ultrafast bootstrap (UFBoot) replicates to assess the final tree topology (Nguyen et al., 2015). For *MADS-box* genes, the best-fit model GTR+F+ASC+R10 was used by IQ-TREE (-pers 0.1, -nm 500) for 10 independent runs. For *TCP* genes, the best-fit model GTR+F+R6 was used by IQ-TREE to infer the phylogeny (default for other options). The consensus tree was annotated and visualized by iTOL (v6) (Letunic and Bork, 2021).

To better identify and classify the Type II type *MADS-box* genes, the K-box domains HMM (PF01486.20), identified by HMMER and curated by SMART, were selected and used, in addition to the MADS domain, for a second round of phylogeny reconstruction. The complete amino acid sequences were first aligned by MAFFT (v7.490) by the FFT-NS-2 strategy. Then, the protein alignment was converted back to codon alignment using PAL2NAL. Further, the residues shared by less than 5% (-gt 0.05) in alignment were trimmed by TrimAl (v1.4.1). In addition, the trimmed alignment was manually curated in Mesquite (v3.61). Finally, IQ-TREE (v1.6.12) was used to infer the maximum-likelihood trees using the GTR+F+ASC+R10 model with 1,000 ultrafast bootstrap (UFBoot) and SH-aLRT test replicates.

### 4.14 Synthesis of gene evolution

The evolutionary history of genes of interest was traced by comparative analysis of the syntenic versus gene sequence relationships by mapping the former on the phylogenetic gene trees. Identified lineage-specific genes within the Asteraceae were then checked for expression in the floral tissues of dandelion to support their diversification. After this, the genes were also checked for their expression in other Asteraceae based on data from the literature.

## Data availability statement

Genome and transcriptome sequence data of T. officinale is deposited in the ENA SRA database under accession nos. PRJEB58885 (genome assembly), PRJEB58886 (RNAseq data of mixed tissues) and PRJEB58887 (25 RNAseq files of floral tissues and 2 of leaves); Supplementary Data S1-S5 (large Excel files) can be found at: https://doi.org/10.4121/22262773.v1.

## Author contributions

KVi, MES, KVe, and IM conceived and designed the project, with previous contributions from JFC. KVi, CO, and MB generated the materials for genome and transcriptome sequencing. JR assembled and annotated the genome, with previous contributions from LB, TZ, and HG. WX performed the phylogeny and synteny analysis. KVi performed the analysis of the expression data. WX, JR, MES, and KVi interpreted the data and wrote the manuscript. All authors critically read and approved the manuscript.
