# Supplementary material for: Phylogenomic analysis provides insights into MADS-box and TCP gene diversification and floral development of the Asteraceae, supported by de novo genome and transcriptome sequences from dandelion (Taraxacum officinale)
Source: Front Plant Sci. 2023 Jun 21;14:1198909. doi: 10.3389/fpls.2023.1198909 (PMC10338227; doi:10.3389/fpls.2023.1198909)
Supplement: Supplementary file 1 [file DataSheet_1.pdf]

Supplementary Material

Phylogenomic analysis provides insights into *MADS-box* and *TCP* gene diversification and floral development of the Asteraceae, supported by *de novo* genome and transcriptome sequences from dandelion (*Taraxacum officinale*)

Wei Xiong[1‡], Judith Risse[2,3‡], Lidija Berke[1†], Tao Zhao[1†], Henri van de Geest[4†], Carla Oplaat[1†], Marco Busscher[1,4], Julie Ferreira de Carvalho[3†], Ingrid M. van der Meer[4], Koen J. F. Verhoeven[3], M. Eric Schranz[1*§], Kitty Vijverberg[1*§]

[1]Biosystematics Group, Wageningen University and Research, Wageningen, The Netherlands, [2]Bioinformatics Group, Wageningen University and Research, Wageningen, The Netherlands, [3]Department of Terrestrial Ecology, Netherlands Institute of Ecology (NIOO-KNAW), Wageningen, The Netherlands, [4]Bioscience, Wageningen University and Research, Wageningen, The Netherlands

***Correspondence:**
Kitty Vijverberg (mail@kittyvijverberg.nl) and M. Eric Schranz (eric.schranz@wur.nl)

Front. Plant Sci. 2023, **14**:1198909. doi: 10.3389/fpls.2023.1198909

Content

Supplementary Figures: 8

Supplementary Tables: 12

Supplementary Data (Excel Files)**:** 5

1 Supplementary Figures

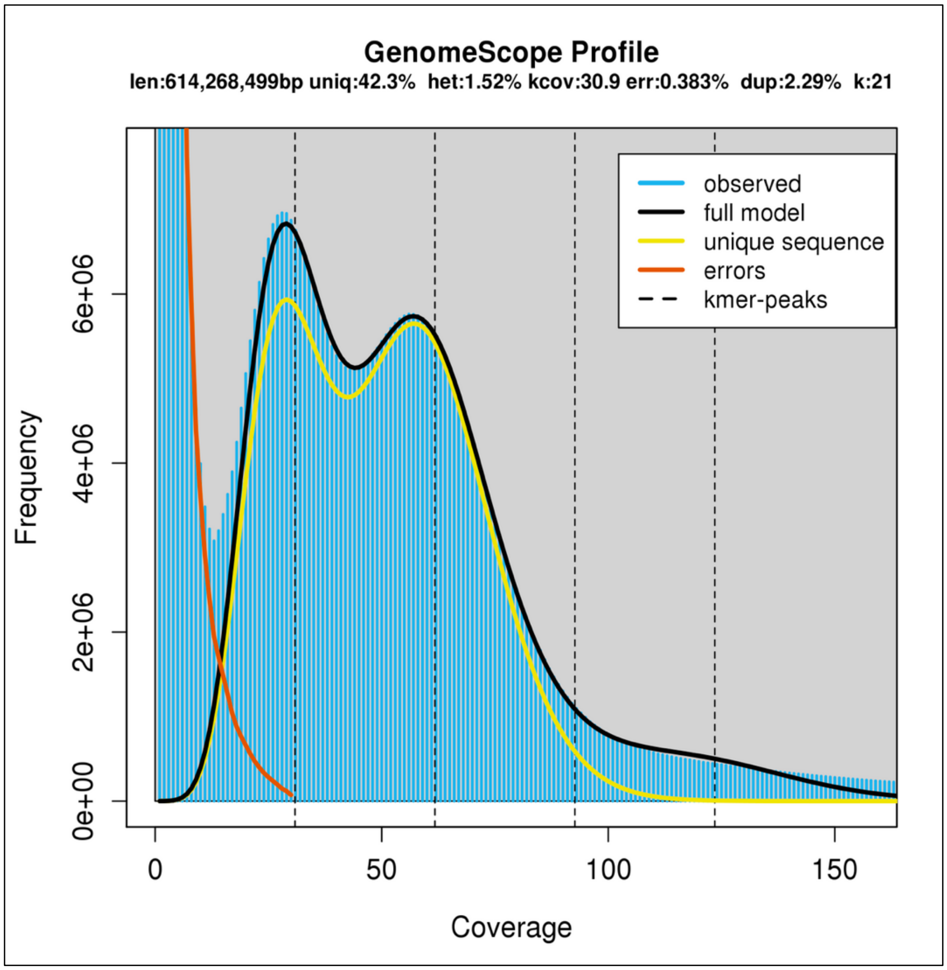

Supplementary Figure S1. Quality profile of the *T. officinal* genome assembly. Kmer (k = 21) analysis showed a low error rate of 0.38% and a relatively high heterozygosity of 1.52%

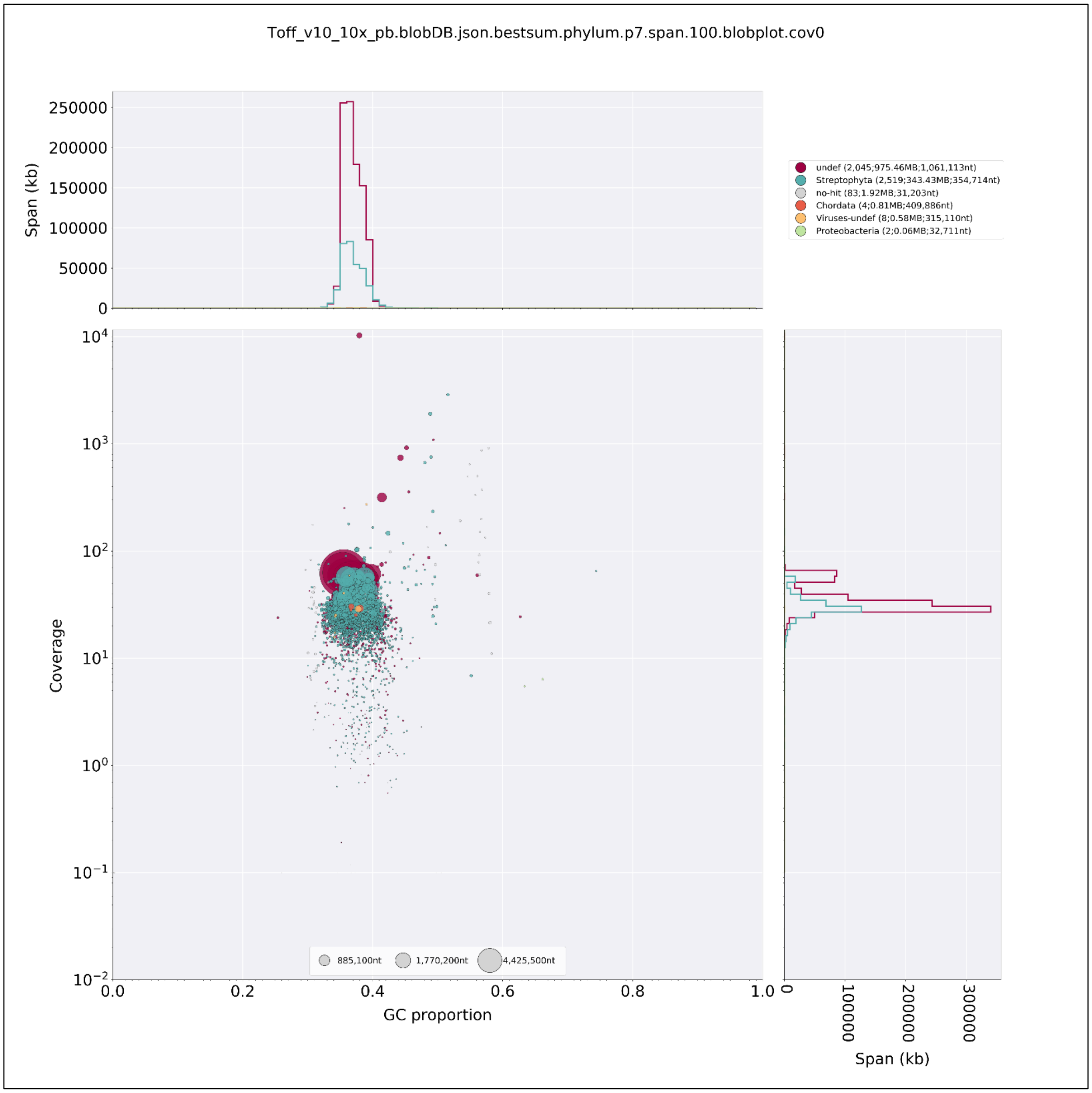

Supplementary Figure S2. Purity of the *T. officinal* genome assembly. Blob Tools analysis indicated a clean assembly, with only very low contamination by microbiome or contaminant organisms (orange, yellow and light green).

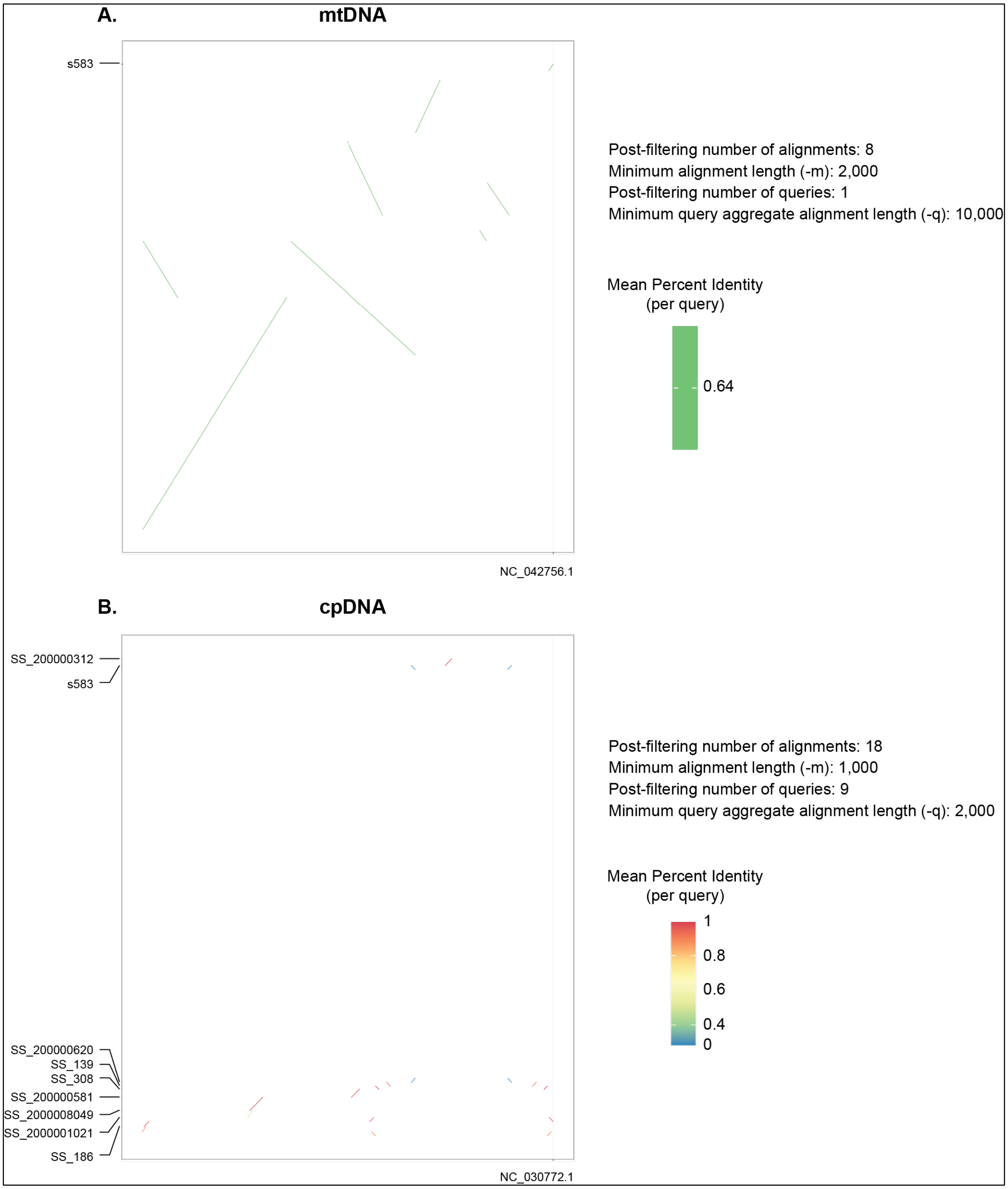

Supplementary Figure S3. Quality of the *T. officinal* plastome assembly. A. The mitochondrial genome (mtDNA) of *T. officinale* was almost entirely assembled in a single scaffold and showed high homology to the mt genome of the related *Lactuca sativa,* with an almost complete coverage and ~65% nucleotide similarity (cultivar Salinas; GeneBank: NC_042756.1). **B.** The chloroplast genome (cpDNA) of *T. officinale* was absent, as is visualized in comparison to the reference cp genome of *T. officinale* (GeneBank: NC_030772), supposedly as a result of bleaching of plants before harvesting tissue for sequencing.

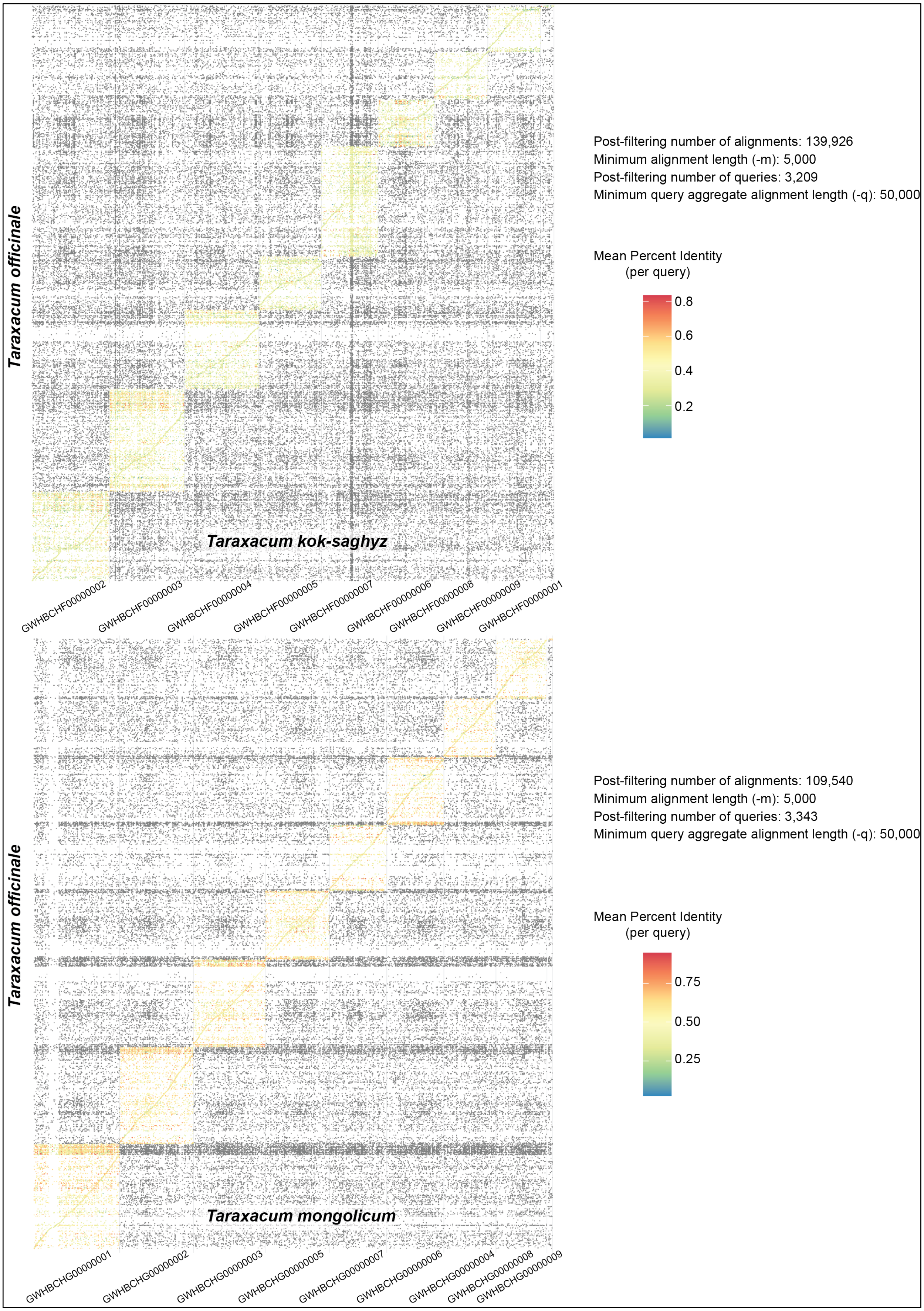
Taraxacum officinale
Taraxacum kok-saghyz
GWHBCHF00000002
GWHBCHF00000003
GWHBCHF00000004
GWHBCHF00000005
GWHBCHF00000007
GWHBCHF00000006
GWHBCHF00000008
GWHBCHF00000009
GWHBCHF00000001
Post-filtering number of alignments: 139,926
Minimum alignment length (-m): 5,000
Post-filtering number of queries: 3,209
Minimum query aggregate alignment length (-q): 50,000
Mean Percent Identity
(per query)
0.8
0.6
0.4
0.2
Taraxacum officinale
Taraxacum mongolicum
GWHBCHG00000001
GWHBCHG00000002
GWHBCHG00000003
GWHBCHG00000005
GWHBCHG00000007
GWHBCHG00000006
GWHBCHG00000004
GWHBCHG00000008
GWHBCHG00000009
Post-filtering number of alignments: 109,540
Minimum alignment length (-m): 5,000
Post-filtering number of queries: 3,343
Minimum query aggregate alignment length (-q): 50,000
Mean Percent Identity
(per query)
0.75
0.50
0.25

Supplementary Figure S4. Genome comparison of *T. officinale* with two other Taraxacum species. Dot blots show the comparison of *T. officinale* to *T. kok-saghyz* (top) and *T. mongolicum* (bottom) (both Lin et al., 2022). The assembly of *T. officinale* is more fragmented than the other two species assembled down to their chromosomes, but showed good co-linearity with both genomes without major structural rearrangements.

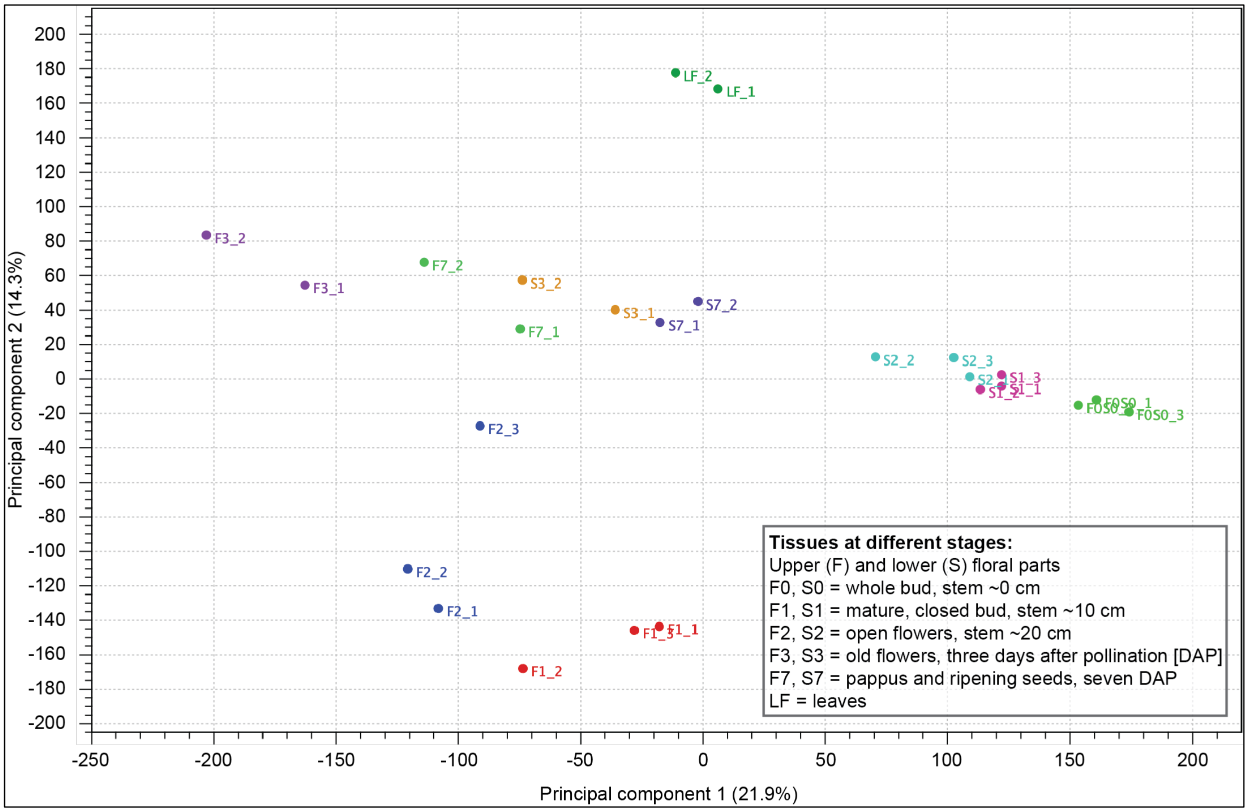

Supplementary Figure S5. Quality control of the *T. officinal* transcriptome data of floral developmental replicates. Principal component analysis showed clustering of replicates per stage and tissue and particularly tight clustering of the youngest stages. See inset and Figure 1C for the tissues analyzed.

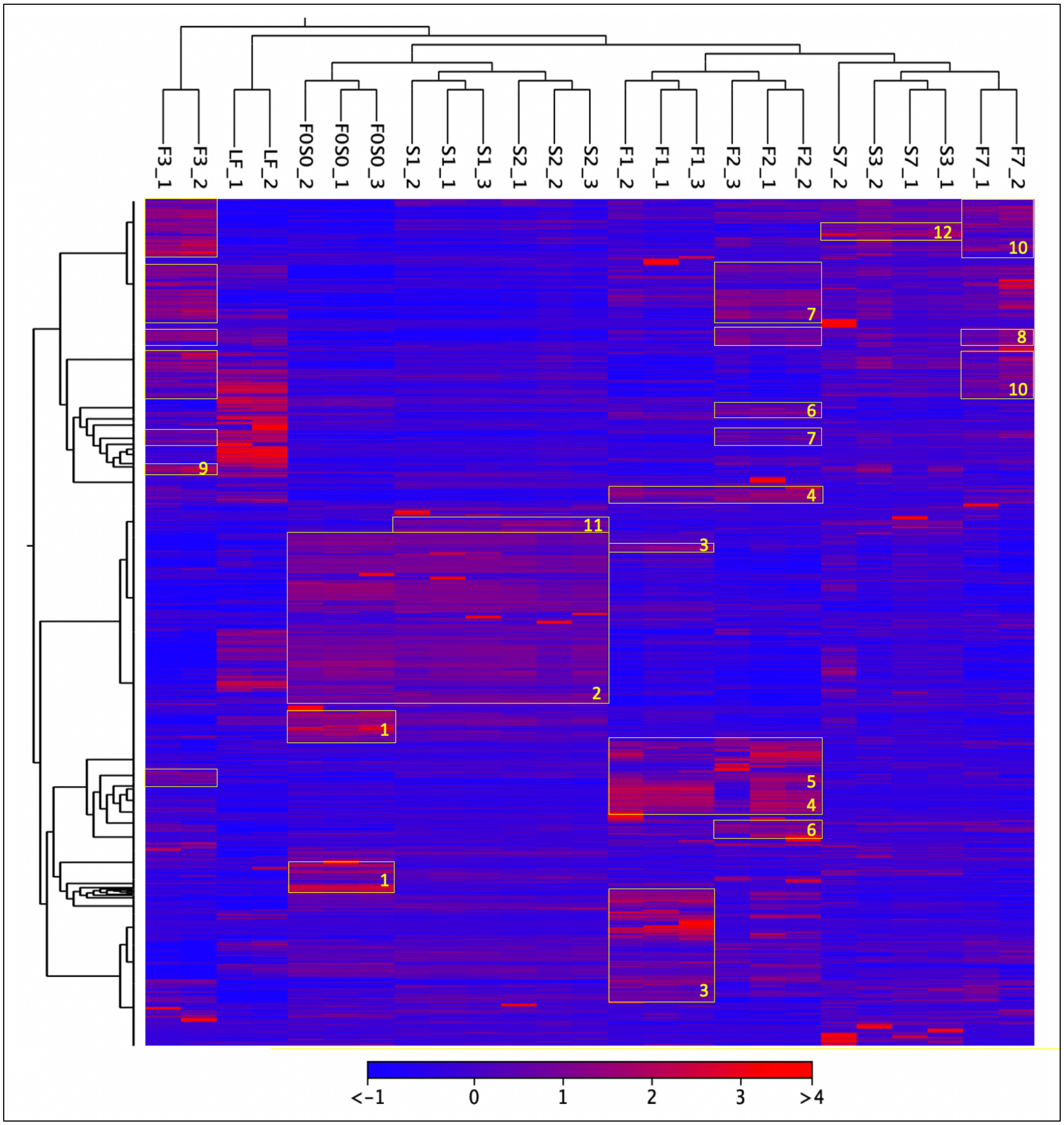

Supplementary Figure S6. Similarity analysis of the *T. officinal* transcriptomes of floral developmental stages and tissues. The heatmap shows all expressed genes (n ~52000, ~60%), confirming a good quality of the data by clustering of samples per replicate, and showed clustering of tissues of subsequent stages in addition, most clearly for the young lower floral parts (S0, S1, S2) and young upper floral parts (F1, F2). White lines box manually defined, tissue specific expression blocks numbers 1-12 (added to Supplementary Data S4, Columns S and T). Expression values are presented in LOG2 (Total Exon Reads [TER] +1, in Transcripts Per Million [TPM]), with red being highest expressed. Stages and tissues analyzed are explained in Figure 1C and the inset of Supplementary Figure S5.

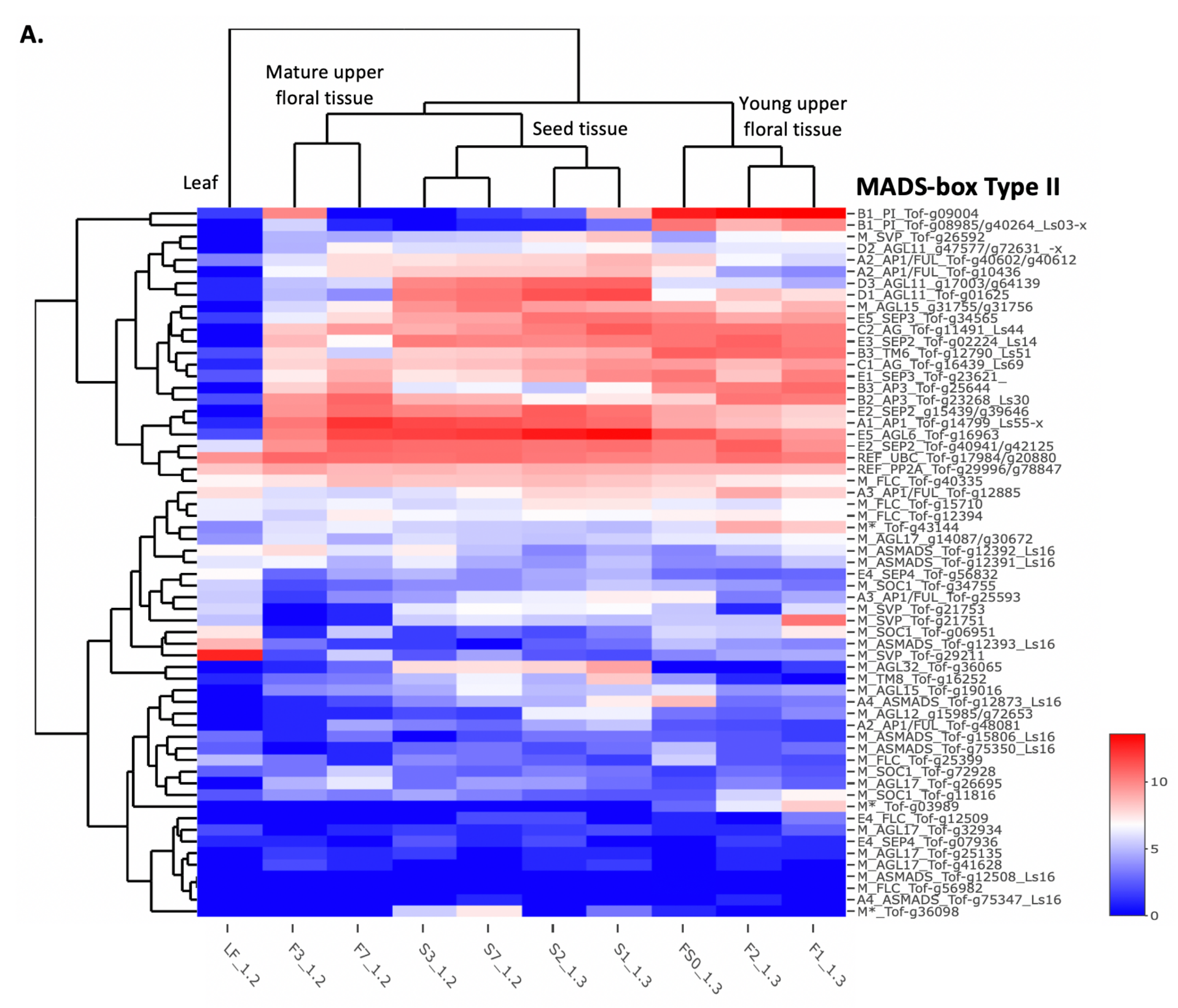
A.
Mature upper floral tissue
Seed tissue
Young upper floral tissue
Leaf
MADS-box Type II
B1_PI_Tof-g09004
B1_PI_Tof-g08985/g40264_Ls03-x
M_SVP_Tof-g26592
D2_AGL11_g47577/g72631_-x
A2_AP1/FUL_Tof-g40602/g40612
A2_AP1/FUL_Tof-g10436
D3_AGL11_g17003/g64139
D1_AGL11_Tof-g01625
M_AGL15_g31755/g31756
E5_SEP3_Tof-g34565
C2_AG_Tof-g11491_Ls44
E3_SEP2_Tof-g02224_Ls14
B3_TM6_Tof-g12790_Ls51
C1_AG_Tof-g16439_Ls69
E1_SEP3_Tof-g23621_
B3_AP3_Tof-g25644
B2_AP3_Tof-g23268_Ls30
E2_SEP2_g15439/g39646
A1_AP1_Tof-g14799_Ls55-x
E5_AGL6_Tof-g16963
E2_SEP2_Tof-g40941/g42125
REF_UBC_Tof-g17984/g20880
REF_PP2A_Tof-g29996/g78847
M_FLC_Tof-g40335
A3_AP1/FUL_Tof-g12885
M_FLC_Tof-g15710
M_FLC_Tof-g12394
M*_Tof-g43144
M_AGL17_g14087/g30672
M_ASMADS_Tof-g12392_Ls16
M_ASMADS_Tof-g12391_Ls16
E4_SEP4_Tof-g56832
M_SOC1_Tof-g34755
A3_AP1/FUL_Tof-g25593
M_SVP_Tof-g21753
M_SVP_Tof-g21751
M_SOC1_Tof-g06951
M_ASMADS_Tof-g12393_Ls16
M_SVP_Tof-g29211
M_AGL32_Tof-g36065
M_TM8_Tof-g16252
M_AGL15_Tof-g19016
A4_ASMADS_Tof-g12873_Ls16
M_AGL12_g15985/g72653
A2_AP1/FUL_Tof-g48081
M_ASMADS_Tof-g15806_Ls16
M_ASMADS_Tof-g75350_Ls16
M_FLC_Tof-g25399
M_SOC1_Tof-g72928
M_AGL17_Tof-g26695
M_SOC1_Tof-g11816
M*_Tof-g03989
E4_FLC_Tof-g12509
M_AGL17_Tof-g32934
E4_SEP4_Tof-g07936
M_AGL17_Tof-g25135
M_AGL17_Tof-g41628
M_ASMADS_Tof-g12508_Ls16
M_FLC_Tof-g56982
A4_ASMADS_Tof-g75347_Ls16
M*_Tof-g36098
10
5
0
LF_1.2
F3_1.2
F7_1.2
S3_1.2
S7_1.2
S2_1.3
S1_1.3
FS0_1.3
F2_1.3
F1_1.3

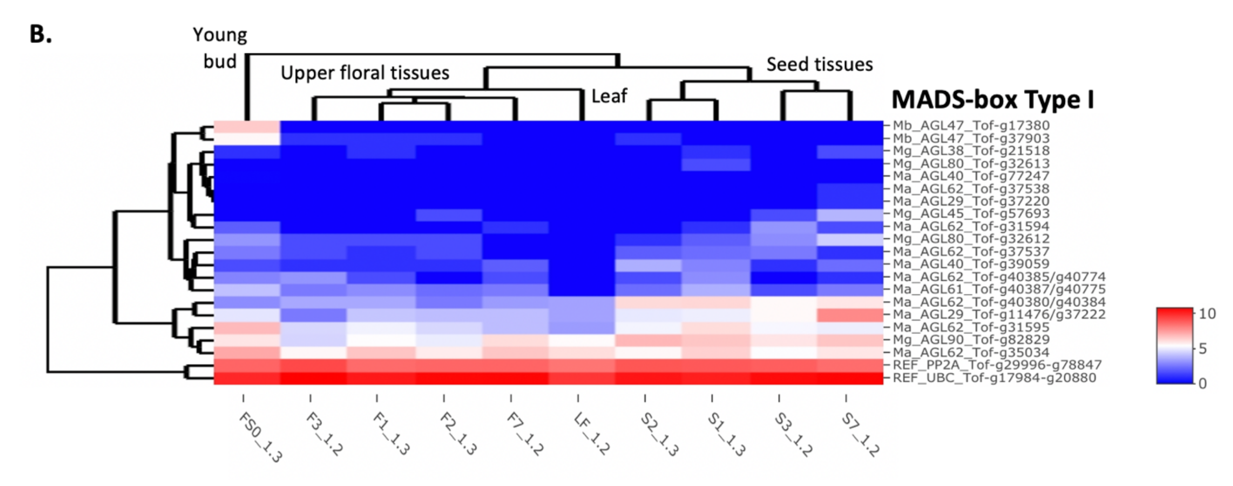
B.
Young bud
Upper floral tissues
Leaf
Seed tissues
MADS-box Type I
Mb_AGL47_Tof-g17380
Mb_AGL47_Tof-g37903
Mg_AGL38_Tof-g21518
Mg_AGL80_Tof-g32613
Ma_AGL40_Tof-g77247
Ma_AGL62_Tof-g37538
Ma_AGL29_Tof-g37220
Mg_AGL45_Tof-g57693
Ma_AGL62_Tof-g31594
Mg_AGL80_Tof-g32612
Ma_AGL62_Tof-g37537
Ma_AGL40_Tof-g39059
Ma_AGL62_Tof-g40385/g40774
Ma_AGL61_Tof-g40387/g40775
Ma_AGL62_Tof-g40380/g40384
Ma_AGL29_Tof-g11476/g37222
Ma_AGL62_Tof-g31595
Mg_AGL90_Tof-g82829
Ma_AGL62_Tof-g35034
REF_PP2A_Tof-g29996-g78847
REF_UBC_Tof-g17984-g20880
10
5
0
FS0_1.3
F3_1.2
F1_1.3
F2_1.3
F7_1.2
LF_1.2
S2_1.3
S1_1.3
S3_1.2
S7_1.2

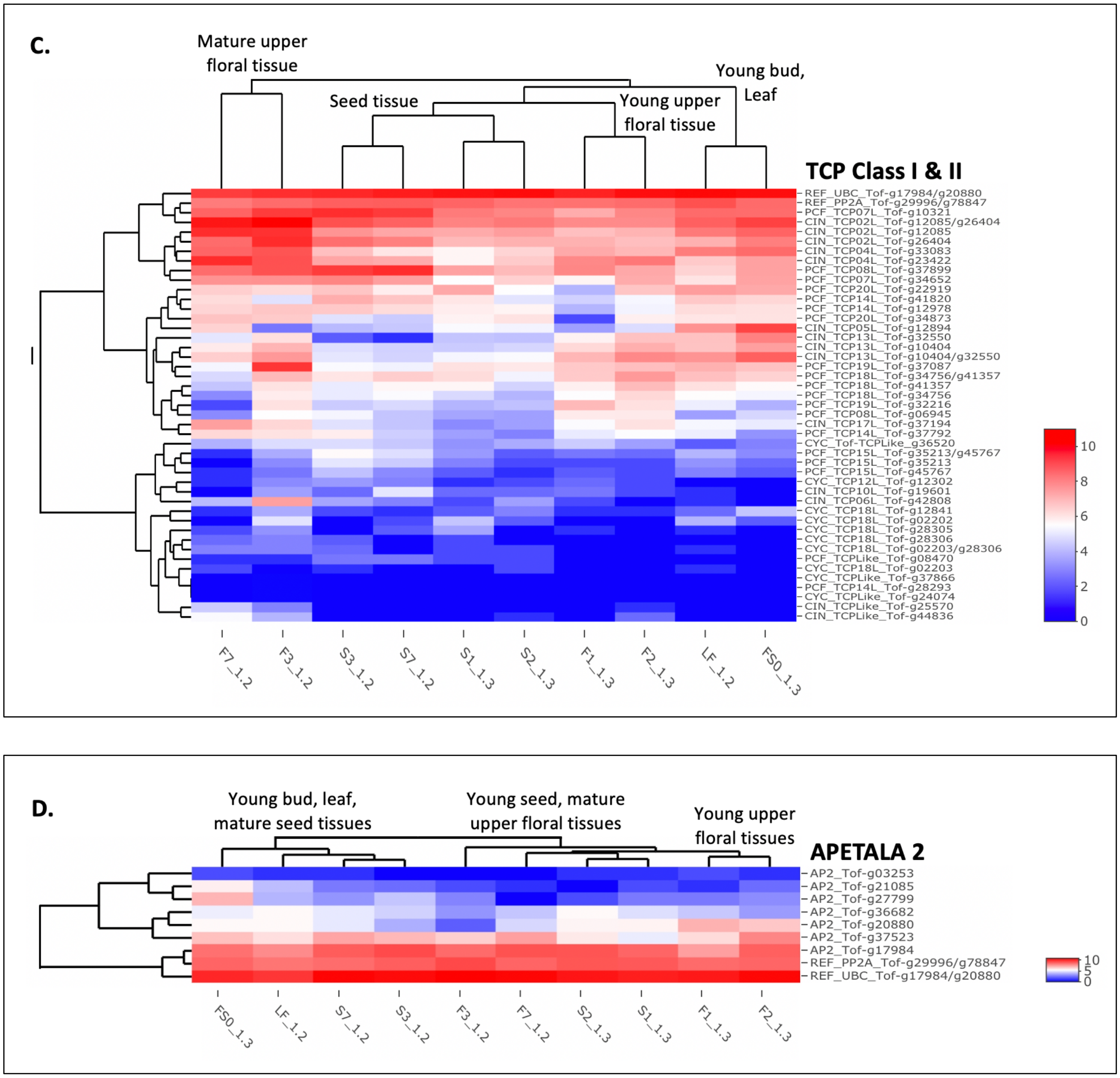

Supplementary Figure S7. Expression of *MADS-box* and *TCP* genes in *T. officinal* floral developmental stages and tissues. The heatmaps show the result of all genes present in *T. officinale* in each of the following four subsets: **A.** Type II *MADS-box* genes, confirming the expression of virtually all members of the ABC(D)E genes in one or more of the floral stages and tissues, while being absent or low expressed in leaves (upper cluster; gene class as a prefix to the names), and the absence or low expression of most other *MADS-box* genes (indicated with a M as prefix). **B.** Type I *MADS-box* genes, showing overall low expression, while confirming relatively high expression of genes know for young ovules (F0S0), lower floral parts (i.e., seeds, S1-7) and/or mature seeds (S7), e.g., *AGL*47 and *AGL*62. **C.** All *TCP* genes, showing expression of the *CIN* genes in all or a subset of floral tissues and stages, while most of the *CYC* genes, known for a role in floral symmetry, are low or unexpressed. **D.** *APETALA*2 genes, the non-*MADS-box* class A genes, confirming their expression particularly in young floral tissues. Expression is presented in LOG2 (average Total Exon Reads [TER] per replicate +1 [in TP10M]), with red being highest expressed. Stages and tissues analyzed are explained in Figure 1C and the inset of Supplementary Figure S5.

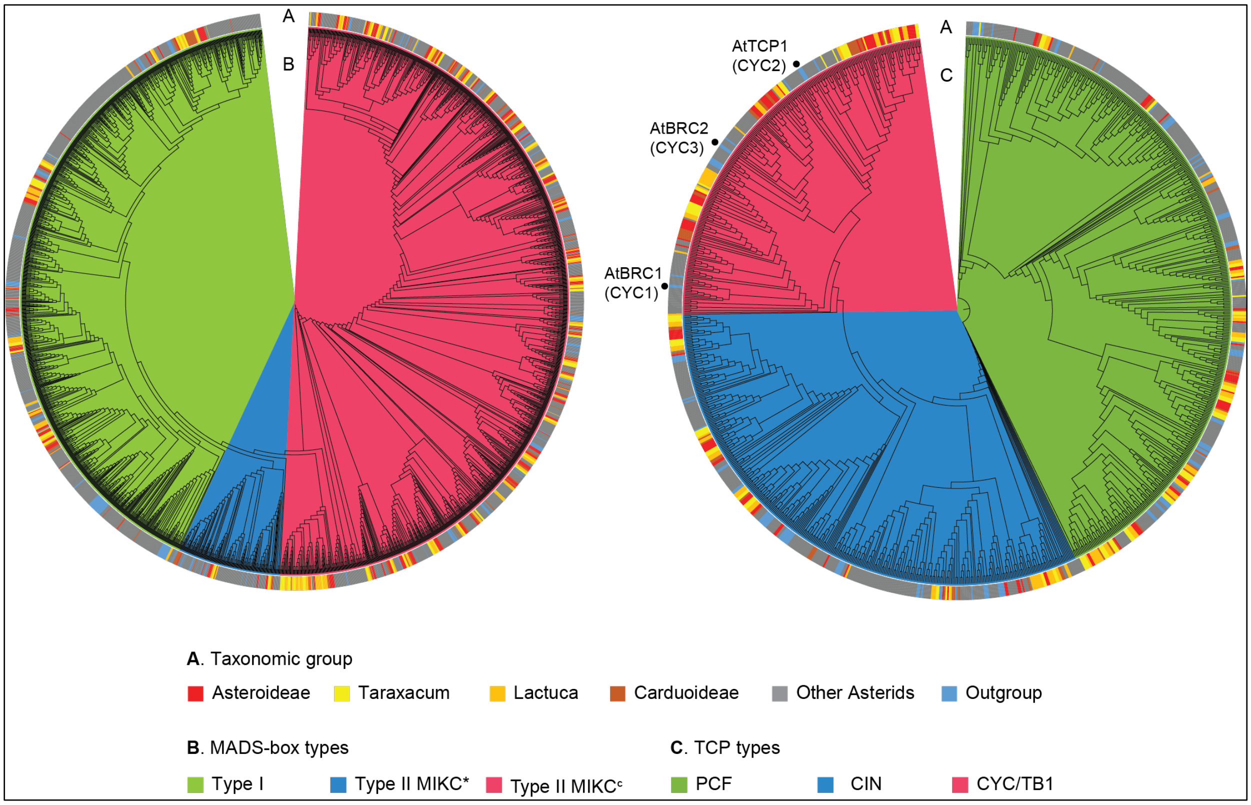

Supplementary Figure S8. Gene trees of *MADS-box* and *TCP* genes. The phylogenetic trees were constructed on the basis of amino acid alignments of the *MADS* domain (left) and *basic HELIX LOOP HELIX* (*bHLH*) domain (right) and included all *MADS-box* and *TCP* genes identified in the 33 species analysed. Subgroups of species are indicated in the outer circles and explained below the threes as well as in Figure 1A; subgroups of genes are indicated in colours within the circles. A phylogenetic tree focused on *MADS-box* Type II genes only, defined on the presence of the *K-box* domain in addition to the *MADS-box* domain, is shown in Figure 1B.

2 Supplementary Tables

Supplementary Table S1.
Statistics of *T. officinale* assembly (based on contigs of size ≧500 bp)

Genome assembly	Contigs	Superscaffold
Assembly size (Mb)	909	936
Number of contigs/superscaffold > 1kb	6,440	4,059
N50 of contig/superscaffold (bp)	288,635	756,557
Longest contig (bp)	6,220,034	22,921,766
Number of scaffolds >50 kb	3,876	2,216
Total length of contigs/scaffolds >50 kb (Mb)	844	891
Sequences in contigs/scaffolds > 50 kb (%)	93	95

Supplementary Table S2.
Genome repeat characteristics of *Taraxacum officinale*

Type	Class	Count	Masked	
			(bp)	(%)
DNA	CMC-EnSpm	4,537	3,022,028	0.32
	MuLE-MuDR	8,465	6,859,711	0.73
	PIF-Harbinger	7,727	4,398,688	0.47
	hAT-Ac	2,509	1,296,404	0.14
	hAT-Tag1	451	323,732	0.03
	hAT-Tip100	3,070	1,365,332	0.15
LINE	CRE-II	841	512,033	0.05
	L1	12,296	14,177,677	1.51
	R1	18	6,078	0.00
	RTE-BovB	181	98,018	0.01
LTR		7,837	3,716,005	0.40
	Caulimovirus	1,402	1,448,505	0.15
	Copia	188,026	214,441,863	22.90
	Gypsy	101,369	135,519,100	14.47
	Pao	39	7,644	0.00
	Unknown	868	37,742,318	4.03
RC	Helitron	3,499	2,668,988	0.29
Unknown		337,288	148,548,843	15.86
**Total**	interspersed	766,355	576,152,967	61.53
Low complexity		2,785	1,438,647	0.15
Simple repeat		210,995	12,757,981	1.36
**Total**		1,005,200	590,349,595	63.04

Supplementary Table S3
Gene prediction statistics of *T. officinale* (based on high quality transcripts of size ≧150 aa with homology annotation)

**Genome annotation**	***Tof* FCh72**
Number of high confident genes	60,810
Number of high confidence transcripts	63,780
Gene density per Mbp	65.5
Mean gene length (bp)	2,110
Mean CDS length (bp)	971
Mean exons per mRNA	4.7
Mean intron length (bp)	306
Transcripts with functional annotation	56,560
Transcripts with functional annotation (%)	88.7
Number of identical transcripts	1,739
Number of identical transcripts (%)	2.7
Number of > 2 identical transcripts	15x4, 4x5
Number of 99% similar proteins	4,788
Number of 99% similar proteints (%)	7.5
Highest gene copy number	15
Second highest copy number	11
Gene with highest copy number	Histone H4
Second highest copy number gene	GOS9-like isoform X1
Transcripts associated to at least on GO term	37,324
Transcripts associated to at least on GO term (%)	61.4
Number of genes with 1 transcripts	58,197
Number of genes with 2 transcripts	3,31
Number of genes with 3 transcripts	257
Number of genes with 4 transcripts	37
Number of genes with 5 transcripts	9
Genes with > 1 transcripts (%)	4.3
Number of genes	81,292*
Number of transcripts	85,093*

*including the smaller genes ≧50 aa, also without homology annotation

Supplementary Table S4
Genome comparison between three Taraxacum species: *T. officinale, T. monogolicum* and *T. kok-saghyz*

**Characteristic**	***Tof* FCh72**	***Tmo* 5**	***Tks* 1151**
	This study	(Lin et al. 2022)	
Ploidy level	**2x**	**2x**	**2x**
Assembly size (Mb)	936	790	1,102
Number of contigs/superscaffold >1kb	4,059	65	160
N50 of contig/superscaffold (kb)	757	96,940	131,570
Longest superscaffold (Mb)	23	130.1	162.5
GC content (%)	37	37	36.9
Repetitive percentage (%)	63	72	76
Heterozygosity (%)	1.5	1.3	~1
BUSCO of assembly* (%)	97.2	93.0	85.6
Number of genes	60,810	45,553	45,224
Number of transcripts	63,780	67,585	56,483

*based on embryophyta_odb10 library containing 1614 BUSCOs (versus eudicots_odb10 library with 2326 BUSCOs used in Table 1)

Supplementary Table S5.
Species and sequences used for Synteny analysis (Separate Excel File)

Supplementary Table S6.
Reference genes for *MADS-box* and *TCP* genes used in this study (Separate Excel File)

Supplementary Table S7.
***MADS-box* and *TCP* gene identification and classification (Separate Excel File)**

Supplementary Table S8.
Read and mapping statistics of transcriptomes of floral tissues at different stages of *T. officinale* plant FCh72 (Separate Excel File)

Supplementary Table S9.
Overall patterns in *T. officinale* plant FCh72 floral transcriptomes (Separate Excel File)

Supplementary Table S10.
***MADS-box* and *TCP gene* expression in floral tissues at different stages in *T. officinale*, averaged per replicate (Separate Excel File)**

Supplementary Table S11. Examples of tandem duplications within the *SFA genes**

Species	Duplicate 1	Duplicate 2	Homolog 1	Homolog 2	Tandem
Beta vulgaris	Bv6_150520_ksif	Bv6_150530_jqqr	*SEP3*	*FLC*	*SEP3_FLC*
Coffea canephora	coc_11_g17100	coc_11_g17110	*FLC*	*SEP3*	*SEP3_FLC*
Chenopodium quinoa	CquiAUR62005643	CquiAUR62005644	*AS-MADS*	*SEP3*	*SEP3_AS-MADS*
Solanum tuberosum	stu_00017759	stu_00017760	*SEP3*	*FLC*	*SEP3_FLC*
Solanum lycopersicum	sly_12g087820.1sly	sly_12g087830.1sly	*AS-MADS*	*FLC*	*AS-MADS_FLC*

**SFA = SEP3 + FLC + AS-MADS*
Supported by Supplementary Data S5.

Supplementary Table S12.
***MADS-box* and *TCP* gene identification and classification (Separate Excel File)**

3 Supplementary Data

Supplementary Data S1.
High confident genes in *Taraxacum officinale*
(Separate Excel File via this link: https://doi.org/10.4121/22262773.v1)

Supplementary Data S2.
a. *MADS-box* genes identification and classification
b. *TCP* genes identification and classification
(Separate Excel File via this link: https://doi.org/10.4121/22262773.v1)

Supplementary Data S3.
a. *MADS-box* Synteny network Clusters
b. *TCP* Synteny network Clusters
c. *MADS-box* Synteny Profiling
d. *TCP* Synteny Profiling
(Separate Excel File via this link: https://doi.org/10.4121/22262773.v1)

Supplementary Data S4.
a. Gene and Transcript expression in floral tissues of *Taraxacum officinale*, Raw data (all transcripts with size >= 150 bp)
b. Gene and Transcript expression in floral tissues of *Taraxacum officinale*, TPM
c. Gene expression in floral tissues of *Taraxacum officinale*, averaged per tissue type, TP10M
(Separate Excel File via this link: https://doi.org/10.4121/22262773.v1)

Supplementary Data S5.
Genome-wide search for *MADS-box* tandem duplicates
(Separate Excel File via this link: https://doi.org/10.4121/22262773.v1)
